# Computational graph pangenomics: a tutorial on data structures and their applications

**DOI:** 10.1007/s11047-022-09882-6

**Published:** 2022-03-04

**Authors:** Jasmijn A. Baaijens, Paola Bonizzoni, Christina Boucher, Gianluca Della Vedova, Yuri Pirola, Raffaella Rizzi, Jouni Sirén

**Affiliations:** 1 Department of Intelligent Systems, Delft University of Technology, Van Mourik Broekmanweg 6, 2628XE Delft, The Netherlands; 2 Department of Biomedical Informatics, Harvard University, 10 Shattuck St, Boston, MA 02115, USA; 3 Department of Informatics, Systems and Communication (DISCo), University of Milano-Bicocca, V.le Sarca, 336, 20126 Milan, Italy; 4 Department of Computer and Information Science and Engineering, University of Florida, 432 Newell Dr, Gainesville, FL 32603, USA; 5 Genomics Institute, University of California, 1156 High St., Santa Cruz, CA 95064, USA

## Abstract

Computational pangenomics is an emerging research field that is changing the way computer scientists are facing challenges in biological sequence analysis. In past decades, contributions from combinatorics, stringology, graph theory and data structures were essential in the development of a plethora of software tools for the analysis of the human genome. These tools allowed computational biologists to approach ambitious projects at population scale, such as the 1000 Genomes Project. A major contribution of the 1000 Genomes Project is the characterization of a broad spectrum of genetic variations in the human genome, including the discovery of novel variations in the South Asian, African and European populations—thus enhancing the catalogue of variability within the reference genome. Currently, the need to take into account the high variability in population genomes as well as the specificity of an individual genome in a personalized approach to medicine is rapidly pushing the abandonment of the traditional paradigm of using a single reference genome. A graph-based representation of multiple genomes, or *a graph pangenome*, is replacing the linear reference genome. This means completely rethinking well-established procedures to analyze, store, and access information from genome representations. Properly addressing these challenges is crucial to face the computational tasks of ambitious healthcare projects aiming to characterize human diversity by sequencing 1M individuals (Stark et al. 2019). This tutorial aims to introduce readers to the most recent advances in the theory of data structures for the representation of graph pangenomes. We discuss efficient representations of *haplotypes* and the variability of *genotypes* in graph pangenomes, and highlight applications in solving computational problems in human and microbial (viral) pangenomes.

## 1 Introduction

The 1000 Genomes Project (The 1000 Genomes Project Consortium 2015) marks the beginning of new computational approaches to genomic studies. The high variation rate among individuals, and the availability of thousands of human genomes have accelerated computational efforts towards graph models as a new paradigm for representing a *reference genome*. The question “what is an ideal reference genome?” is becoming the focus of investigations that also involve theoreticians in the computer science community. In this direction, algorithmic approaches have been proposed to implement pangenome graphs. Moreover, the literature presents experimental evidence of the advantages of those approaches (Rakocevic et al. 2019; Sibbesen et al. 2018; Dilthey et al. 2015; Garrison et al. 2018). Various reviews have presented this new research field (Paten et al. 2017; Eizenga et al. 2020b), while challenges from different domains are outlined by Computational Pan-Genomics Consortium (2018).

The aim of this tutorial is to discuss the main algorithmic approaches and issues that will represent the focus of computer science research in the next years. After illustrating the motivation for computational pangenomics, the tutorial discusses recent succinct data structures that are highly promising in main applications of pangenomics. The tutorial is organized as follows. First, the basics of computational pangenomics are presented, including construction of a pangenome graph, possible graph representations, operations over a pangenome, and data structures that index a pangenome. Second, related to this last concept, we present recent data structures in pangenomics, the *positional* Burrows–Wheeler Transform and its generalization to manage graphs, called *graph BWT*. Third, issues related to time and space complexity are addressed by illustrating the essentials of the *r*-index based data structure that allows efficient implementation of well known queries, such as finding maximum exact matches (MEMs). Lastly, we conclude with exemplifications of the uses of the above mentioned methods to application scenarios aimed at detecting and representing pangenome variation such as in haplotyping and genotyping computational problems. A final section is devoted to the discussion of open problems.

## 2 From a linear sequence to a graph reference of a genome

The term *pangenome* goes back more than fifteen years ago to the framework of microbial analysis of the entire genomic repertoire of a given phylogenetic clade (Tettelin et al. 2005). A pangenome describes the union of sequence entities, such as genes or open reading frames, shared by genomes of a clade. Its main purpose is to represent commonly present and frequently absent sequences (e.g., genes) of interest. While the word “pangenome” in the microbiology literature is often used to describe core genes and strain specific genes, pangenomics is becoming the conceptual framework to deal with the trends in genomics of the last decade: the extraordinary growth of information on human genomes, and the discovery of significant levels of large-scale genomic variation in many eukaryotic species.

In contrast to a linear-genome reference, a pangenome is a reference system for representing sequence variations of the genomic sequence of a species. In particular, a *pangenome graph* is conceived to be the ideal representation for a variety of bioinformatics tasks, which were originally performed on a linear reference genome. This graph encodes the commonalities and differences among a collection of genomes of the same species at the sequence level. The interest in replacing linear reference genomes with pangenome graph models has largely increased with the discovery of limitations in performing various tasks, such as read mapping and variant calling.

### 2.1 Limitations of a linear reference genome

**Fig. 1:**
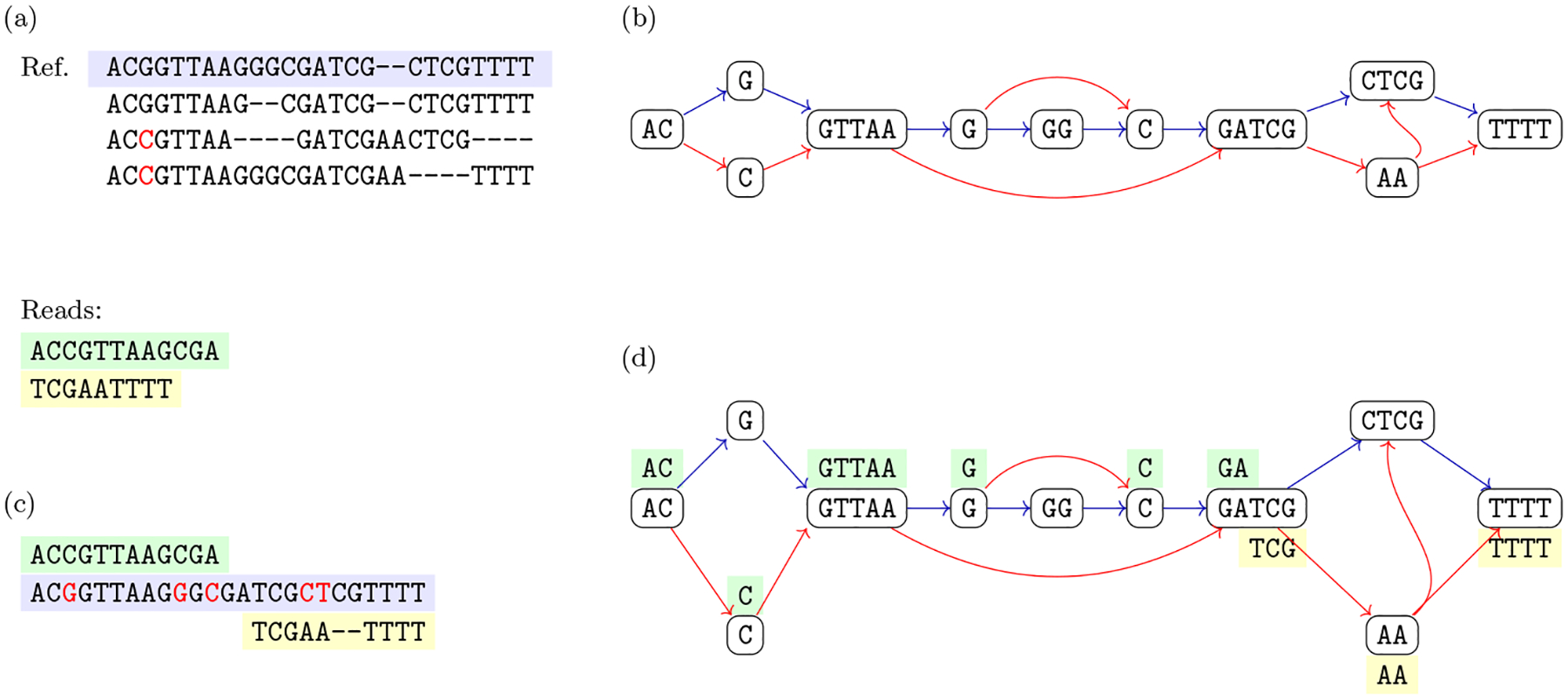
A toy example of how a pangenome graph improves the quality of mapping reads to a reference genome. **a** A multiple sequence alignment of a linear reference genome and other three genomes that contain variations w.r.t. the reference. **b** A variation graph built from the matrix of the multiple alignment of the genomes; in red the edges that represent variations in the graph and form the typical “bubbles” in the graph. Observe that the graph may contain a path that does not represent any input genome (for example, ACCGTTAAGGGCGATCGAACTCGTTTT). **c** Mapping of two reads (ACCGTTAAGCGA and ACCGTTAAGCGA) to the linear reference genome. Observe that the alignments induces mismatches and indels. **d** Mapping of the same reads to the variation graph. Observe that, in this case, the mapping is possible without any mismatch

Conventionally, a structural variant (SV) is a genomic mutation involving 50 or more base pairs. SVs can take several forms such as deletions, insertions, inversions, translocations, or more complex events. The study of the 1000 Genomes Project with short reads technologies has enabled the discovery of more than 88 million variants of variable length—84.7 million single nucleotide polymorphisms (SNPs) and 3.6 million short insertions/deletions (indels)—and 60,000 structural variants. On the other hand, it is estimated that the typical genome contains about 2500 large SVs in total, and one SNP every 1200 to 1450 bases (The 1000 Genomes Project Consortium 2015). The introduction of accurate long read sequencing technology to the detection of SVs revealed an even larger number of candidate variations in an individual genome w.r.t. the reference genome (Khorsand et al. 2021). The discovery of so many variants has shed light on major limitation of linear references: reads sampled from an individual carrying certain SVs may not align to the reference—in which case, the read is frequently considered an artifact and discarded. Moreover, the presence of rare alleles in the reference introduces a bias when mapping reads (see Fig. 1). Since mapping reads is still a crucial step in most analyses for the identification of genetic variants that are linked to disease, clinical applications need to go beyond the linear reference genome.

Ballouz et al. (2019) identified other limitations of a linear reference, such as the difficulties in introducing changes in the current reference, and the fact that it does not sufficiently capture population diversity. A reference genome is often thought of as a healthy baseline, while it is not a *healthy* genome, nor the most common, nor the longest, nor an ancestral haplotype. Moreover, there are some clear advantages in using a pangenome reference (Ballouz et al. 2019): reducing reference bias, increasing mapping accuracy when sequencing a new individual (Rakocevic et al. 2019), increasing rare variant identification accuracy, and improving de novo assembly of a new individual. At the same time, representing population diversity is essential in genome-wide association studies for precision medicine (Popejoy and Fullerton 2016). Approaches based on linear reference genomes underlie a particular consensus model of the genome which is convenient but not fully realistic. When using such a model, reconstructed genomes are often more similar to the reference than they actually are (Rakocevic et al. 2019).

A reference genome stored as a linear sequence would fail in representing the diversity in the human population—ignoring the need to represent the diversity, for example, in the African population, which has been traditionally under-represented in biomedical research. In 2016, Popejoy and Fullerton (2016) state that 81% of the genome-wide association study data were from European ancestry, with the other percentage mainly given by Asian populations. Moreover, African populations, which show high variability, are not captured in association studies (Choudhury et al. 2020a). The fact that a single donor of admixed African and European ancestry has contributed the majority (more than 70%) of the current human reference genome (Schneider et al. 2017; Green et al. 2010), the known GRCh38, is a clear limitation since a single individual cannot be representative of the variability in a large population. The above observation that the majority of DNA in the reference from the human genome project is likely to come from African-American ancestry is also confirmed by the evaluation study of rare reference alleles (RRA) by Magi et al. (2015), where it is shown that more than 25% of GRCh38 RRAs are only found in African populations of the 1000 Genomes Project, while 4% are European, 2.1% are Asian, and 1.1% are American. Consequently, more variation will be missing from the reference genome in cohorts with higher diversity (African populations) and drift from donors (East Asian) who provided material for it and with lower diversity. It is expected that even a larger number of variations will be incorporated into the reference genome with the expansion of several ongoing sequencing projects.

At the same time, the development of approaches relying on linear genomes is well consolidated. For instance, the Variant Call Format (VCF) (Danecek et al. 2011) has been widely adopted by the scientific community as the core file format to represent the information of a collection of multiple genomes. This format allows for the representation of relatively simple variations that can be easily reconciled with a linear reference: insertions, deletions, and nucleotide mutations called *single nucleotide polymorphisms* (SNPs).

### 2.2 Graph representations for multiple genomes

Graphs have been extensively used in the literature to model genome sequences. Assembly graphs (i.e., de Bruijn graphs (Compeau et al. 2011) and string graphs (Myers 2005)) are the most well-known type of graph used to store and represent biological data. These graphs are built from fragments of a genome which are commonly referred to as *sequence reads*, and represent the common regions between reads (fixed or of variable length) as edges in the graph. These graphs will be discussed in detail in Sect. 6.2. Sequence reads are produced by sequencing technologies and have different characteristics in terms of length, errors and throughput, meaning the amount of data that can be produced in a single run of the machine.

Overlap graphs form a specific type of string graphs, where vertices represent sequence reads and arcs indicate non-empty overlap (either exact or inexact) between the reads reads (Rizzi et al. 2019). In particular, string graphs (Myers 2005), introduced to assemble genomes from sequence reads, provide a graph representation of genome sequences with some features that are especially useful: (1) each vertex is labeled by a sequence and its reverse-complement, (2) arcs connect two sequences that appear consecutively in the genome (possibly with an overlap), and (3) walks correspond to portions of the genome.

Assembly graphs introduce another complication, since we cannot know the strand from which the read has been extracted. In this case, each vertex has two labels, where one is the reverse complement of the other. As customary for assembly graphs, we represent only the canonical label—the label that is lexicographically smaller – but each walk must distinguish between the two labels. Partially ordered graphs (Lee et al. 2002) have also been used to represent the sequence alignment of multiple genomes. This is one of the first approaches used for representing shared sequences among multiple genomes. Partially ordered graphs have been investigated in the literature and at the same time some graph representations have been proposed to store multiple sequences or assembly graphs (Li et al. 2017).

### 2.3 Pangenome graphs and their main applications

Pangenome graphs have been proposed as a new paradigm for representing reference genomes. This is a natural representation since graphs provide a compact and concise data structure for performing several tasks, including classical search operations. Graph-based representations of the human genome may encode a large number of variants, such as those reported by The 1000 Genomes Project Consortium (2015). However, the size and number of such graphs is likely to further increase with the completion of ongoing sequencing projects. The adoption of pangenome graphs in performing tasks for the analysis and comparison of genomes in presence of variations is only at the beginning, but such pangenomics approaches have shown to outperform single reference genome approaches.

- *Structural variant graph representation* is a computational problem that is relevant for many tasks. It is not possible to represent complex structural variants with use of a single reference genome. Structural variants may change a genome into a similar but functionally different genome, and are the result of rearrangements of sequence segments in the genome, such as for example the duplication, inversions and translocation of segments of the genome. A graph is a more appropriate structure to represent rearrangements among multiple genomes, since orientation of edges, cycles and complex structures in a graph, such as *bubbles*, represent structural variants in a way that they can be managed by algorithms and suitable data structures to index and query graphs. A bubble is a directed acyclic subgraph determined by a pair of vertices, a source vertex *s* and a terminal vertex *t* such that all paths from *s* to *t* are vertex disjoint.
- *Highly accurate read alignment* to regions of high variability. Read alignment to a sequence is the operation of establishing the location in the sequence where the read originated as a fragment. There are regions in the human genome that are important for immunology studies but very challenging for read alignment due to the large number of variations. An example is given by the ~5 million base region in the human genome called the Major Histocompatibility Complex (MHC). Providing a suitable pangenomic representation for read alignment—especially within these regions of the human genome—is an important computational challenge.
- *Genotyping variants* is the problem of reconstructing the allele variants that characterize an individual. Due to the diploid nature of the human genome, chromosomes come in pairs that are highly similar but present differences at the nucleotide level. For example, nucleotide differences can occur, and determine the homozygous or heterozygous state of positions or loci of the chromosomes: homozygous loci bear the same value on both chromosome copies, while heterozygous loci bear different values on the two copies. Genotyping an individual is a computational task that is performed by having as input a sample of reads from the individual (Denti et al. 2019). Typical genotyping approaches make use of read alignment to a linear reference, in which case SVs or any main difference at the sequence level between the reference and the individual sample may potentially lead to bias and erroneous and incomplete genotyping.
- *Haplotype resolved pangenome analysis* is a computational task aiming to specify haplotype information in a graph representation. While genotyping an individual means to specify the fact that a site is homozygous or heterozygous, haplotyping (or *phasing*) of the genome consists in determining on which chromosomal copy, *i.e*., paternal or maternal, the different alleles are located (Bonizzoni et al. 2016).

It is interesting to note that solving the problem of genotyping variants means combining some of the above listed tasks, starting from a suitable representation of highly polymorphic regions and finally considering the alignment of reads to that representation. Giraffe (Sirén et al. 2021) is a recent approach based on short read alignment for genotyping of SNPs, indels, and SVs genome-wide. Highly polymorphic or repetitive regions represent a challenge for SV prediction tools due to the fact that a linear reference model is unable to capture the complexity of such information. Genotyping tasks are usually performed by mapping of reads: this is a task which is very fast in BWA-MEM (Li 2013) on a single linear reference, but it may be slower on a graph. Giraffe is a fast mapper of short reads to a pangenome graph consisting of aligned haplotypes indexed by the graph BWT described in one of the next sections. An important ingredient for read alignment to a pangenome in Giraffe is the ability to efficiently match queries over the graph by the graph BWT.

In Sect. 6 we will detail two main application scenarios of the concepts presented in the following sections.

### 2.4 On the structure of the paper

First, we will focus on formally introducing the definition of sequence graphs and variation graphs. Indeed, to the best of our knowledge, the literature does not present a widely accepted formal definition of variation (or sequence) graphs: most of the papers either have a focus on graphs, where the labels of the vertices are almost neglected (for example, Paten et al. 2017), or the focus is on strings and the graph is implicit (see Ukkonen 2002; Huang et al. 2013). One of the few papers that considers a notion of variation graph similar to the one we propose in the tutorial is presented by Sirén (2017), but the focus of that paper is on indexing graphs. For this reason, we focus on defining variation graphs. Secondly, we discuss relevant computational problems, such as:

- How to define a pangenome graph and inspect its properties,
- How to build a pangenome graph from a collection of genomes,
- How to store a pangenome graph and index the information contained therein, so that reads can be efficiently mapped to the pangenome.

Despite the fact that computational pangenomics is in its early stages, several competing and/or complementary approaches have been proposed, such as VG (Garrison et al. 2018), SevenBridges (Rakocevic et al. 2019), PaS-GAL (Jain et al. 2019), GraphAligner (Rautiainen et al. 2019), and odgi (Guarracino et al. 2021). Next, we describe some data structures and algorithms that can index pangenomes techniques. In particular, we present the positional BWT, the graph positional BWT, and the *r*-index. We show how the positional BWT allows to store and query in compact space a collection of haplotype sequences. The graph BWT is a generalization of the positional BWT that allows to store the structure of a pangenome graph, the *r*-index leverages the high similarity of multiple genomes to generate in a scalable way to index collections of genomes. These aspects require us to also give a brief introduction of the BWT and the FM-index.

We proceed with an important application of the notions discussed in this tutorial: viral haplotype reconstruction, where we want to build the pangenome of different viral strains.

Finally, we conclude the paper with a discussion of the limitations of the current state of research in computational pangenomics and we provide some open problems.

To simplify the presentation, we assume that the reader is familiar with the basic terminology on graphs (Diestel 2005).

## 3 Pangenome graphs: basic definitions

Given a collection of genome sequences, a fundamental computational problem in pangenomics is how to construct a graph that summarizes the genomes. In this tutorial, a variation graph is vertex-labeled, and some of its paths correspond to the sequences that we want to encode (Garrison et al. 2018). The next two definitions synthesize those that have appeared in literature.

**Definition 1** (*variation graph*) A *variation graph G* = 〈*V*, *A*, *W*〉 is a directed graph whose vertices are labeled by nonempty strings, with *λ* : *V*↦Σ^+^ being the labeling function, and where *A* denotes the set of arcs and *W* denotes a nonempty set of distinguished walks.

In Definition 1 walks correspond to variants (i.e., sequences) that we want to retain in our representation. We note that the set of variants is not explicitly known in some applications, and we want to represent the variants that are compatible with a set of sequence variations. This leads to the definition of sequence graphs (Rakocevic et al. 2019). Sequence graphs represent the set of walks of a variation graph but since these walks are not explicitly labeled, i.e., distinguished, also variants not in the input set which are induced by the arcs of the variation graph are represented (see Fig. 1 for an example of a variant represented in the graph but not in the input genomes).

**Definition 2** (*sequence graph*) A *sequence graph G* = 〈*V*, *A*〉 is a directed graph whose vertices are labeled by nonempty strings, with *λ* : *V*↦Σ^+^ being the labeling function, and where *A* denotes the set of arcs.

**Fig. 2:**
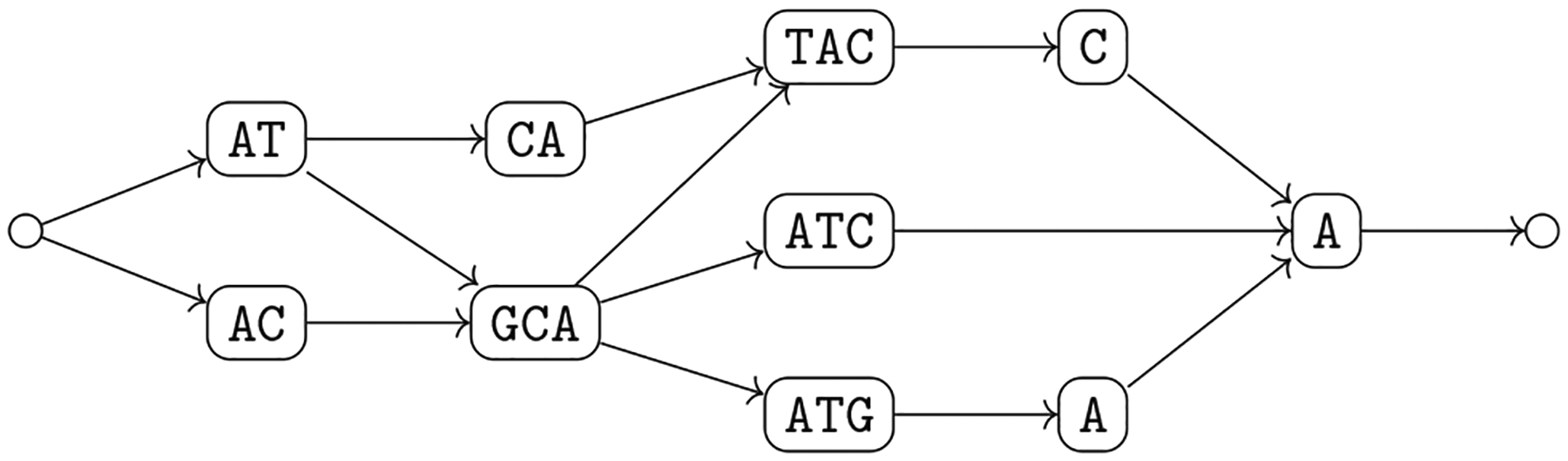
Example of a variation graph with two dummy vertices: a source and a sink

We note that a sequence graph *G* = 〈*V*, *A*〉 is a variation graph *G* = 〈*V*, *A*, *W*〉 with the same set of vertices with *W* consisting of all possible walks in the graph. For this reason, the properties of variation graphs also hold for sequence graphs. To follow the usual nomenclature that is based on the notion of a path, we will mostly use the term “path” even when we refer to a walk. To simplify the exposition, we assume that have a source and a sink of the graph, which are unlabeled (see Fig. 2). Moreover, we make the assumption that a variation graph models a single chromosome. A distinct variation graph for each chromosome for modeling genomes with multiple chromosomes. Next, we note that we can extend the definition of label of a vertex to define also the label of a path. This essentially requires that an arc connects two non-overlapping strings; in this case the graph is *blunt* (Eizenga et al. 2021).

**Definition 3** (*path label*) Let *G* be a variation graph, and let *w* = <*v*_1_, *e*_1_, …, *v*_*l*_> be a walk of *G*. Then the *label* of the walk *w* is the concatenation *λ*(*w*) = *λ*(*v*_1_) ⋯ *λ*(*v*_*l*_) of the labels of the vertices of the walk.

**Definition 4** (*expresses*) Let *g* be a string, and let *G* be a variation graph. Then *G expresses g* if there is a source-sink walk *w* of *G* such that the label of the walk *w* is exactly *g*, that is *λ*(*w*) = *g*.

The definition of a variation graph that we have provided is simple and can be adapted to different contexts. In the case where we want to represent a set of genomes, the variation graph is called a *genome graph* (Eizenga et al. 2020b). A variation graph can be used also to represent an assembly graph – albeit for assembly graphs built from sequencing reads, more specialized and efficient representations are used.

We can consider a variation graph as an abstract data structure for which some concrete implementations have been proposed (Eizenga et al. 2020a). Those implementations present different trade-offs. For example, not all of them easily allow updates in the variation graph, i,e., use dynamic data structures. Moreover, they use different compression strategies and also store strands, to allow a vertex to represent two reverse-complemented strings. We describe a slightly simplified model, where two reverse-complemented strings are represented with two vertices that are linked together, e.g., by sharing an identifier for the pair. The first implementation, VG (Garrison et al. 2018), uses a hash table to represent arcs, but this requires too much memory. A second implementation, XG (Garrison 2019), instead is static, meaning the vertices and arcs cannot be updated. It uses bitvectors to encode the vertices and the adjacency lists, resulting in a fast and memory efficient structure. The third implementation, odgi (Guarracino et al. 2021), represents arcs and walks via delta encoding, where only the difference between the identifiers of two consecutive vertices are stored. Observe that when the graph is similar to a single walk (which is true in almost all practical cases), this encoding couples a great runtime performance with a small memory usage.

A more practical problem is how to store a pangenome graph in a file. The most widely used format for this purpose is GFA, which was initially proposed for representing assembly graphs (Li et al. 2017). It is a textual format to represent labeled graphs. The main limitation of GFA stems from its original purpose. Since an assembly graph has no direct connection with the linear reference genome, a GFA file is not guaranteed to provide a coordinate system that is valid for the entire graph. To overcome this problem, an extension, called rGFA (Li et al. 2020), has been proposed, where a reference walk is selected and determines a coordinate system for the walk. Then each vertex of the graph is associated with a vertex of the reference walk to obtain a coordinate system for the entire graph. In other words, rGFA only considers walks corresponding to simple variants of the reference walk, i.e., cycles in the graph are not allowed. We note that other approaches that provide a coordinate system based on the set of paths exist, for example odgi (Guarracino et al. 2021). While being a clear improvement on the previous methods, odgi has two limitations: the coordinate of a vertex belonging to two different walks is not intuitive, and a vertex that does not belong to any of the walks in *W* has no coordinate. Overcoming these two limitations is a theoretical challenge and the overall notion of coordinate system is still worthy of further investigation.

### 3.1 The construction of a pangenome graph from multiple genomes

A basic problem in computational pangenomics is to build a variation graph. This problem comes in two flavours, depending on whether the input is a set of sequences (corresponding to walks of the graph), or a multiple alignment of the sequences. The latter problem is easier but the quality of the graph is highly dependent on the method used to build the alignment. Since we want to find a variation graph that is able to represent one or more genomes, we need to formally define this notion of representation. Notice that, constructing such a variation graph can be seen as a two-step process: first, we compute a sequence graph representing the genomes, and then we extract the set of walks expressing the genomes.

It is immediate to note that there can exist more than one variation graph expressing a given set of genomes, and some of these graphs do not resemble an alignment, e.g., they might contain a cycle. While we refer the reader to Gusfield (1997) for a more detailed exposition of multiple sequence alignments, in our context, given a sequence *s* = *s*_1_*s*_2_ ⋯ *s*_*l*_ an *aligned* sequence *t* is obtained from *s* by inserting gaps, where a gap is a string made of the character −. An alignment of a set of sequences consists of a set of equal-length aligned sequences, one for each input sequence. Moreover, given two strings *s*_1_ and *s*_2_ we write $S_{1}≙S_{2}$ if removing all gaps from *s*_1_ and *s*_2_ results in the same string.

**Definition 5** (*compatible with an alignment*) Let $G=\left\{g_{1},\ldots ,g_{m}\right\}$ be a set of *m aligned* genomes, all of length *n*. Let *G* = 〈*V*, *A*, *W*〉 be a variation graph that expresses all genomes in G. Then *G* is compatible with the alignment G if there exists:

1. a set *I* of disjoint intervals covering [1, *n*], that is (a) given two intervals [*b*_1_, *e*_1_] and [*b*_2_, *e*_2_] of *I*, either *b*_1_ > *e*_2_ or *b*_2_ > *e*_1_, and (b) for each integer *i* between 1 and *n* there exists an interval [*b*, *e*] ∈ *I* such that *b* ≤ *i* ≤ *e*.
2. a surjective function *ϕ* : *B*↦*V* where *B* is the set of *blocks*, that is the set of pairs (*g*, [*b*, *e*]) with g∈G, [*b*, *e*] ∈ *I* and the string *g*[*b* : *e*] does not consists of only a gap, such that:
  - λ(ϕ(g,[b,e]))≙g[b:e],
  - given the sequence 〈*c*_1_, …, *c*_*k*_〉 of blocks corresponding to the aligned genome *g*, the sequence 〈*ϕ*(*c*_1_), …, *ϕ*(*c*_*k*_)〉 of the vertices associated to such blocks is a walk of *G*;
  - for each arc (*v*, *w*) ∈ *A*, there exist two blocks (*g*, [*b*_1_, *e*_1_]), (*g*, [*b*_2_, *e*_2_]) ∈ *B* with *e*_1_<*b*_2_, *ϕ*((*g*, [*b*_1_, *e*_1_])) = *v*, *ϕ*((*g*, [*b*_2_, *e*_2_])) = *w* and such that there does not exist another block (*g*, [*b*_3_, *e*_3_]) ∈ *B* with *e*_1_<*b*_3_<*e*_3_<*b*_2_.

**Fig. 3:**
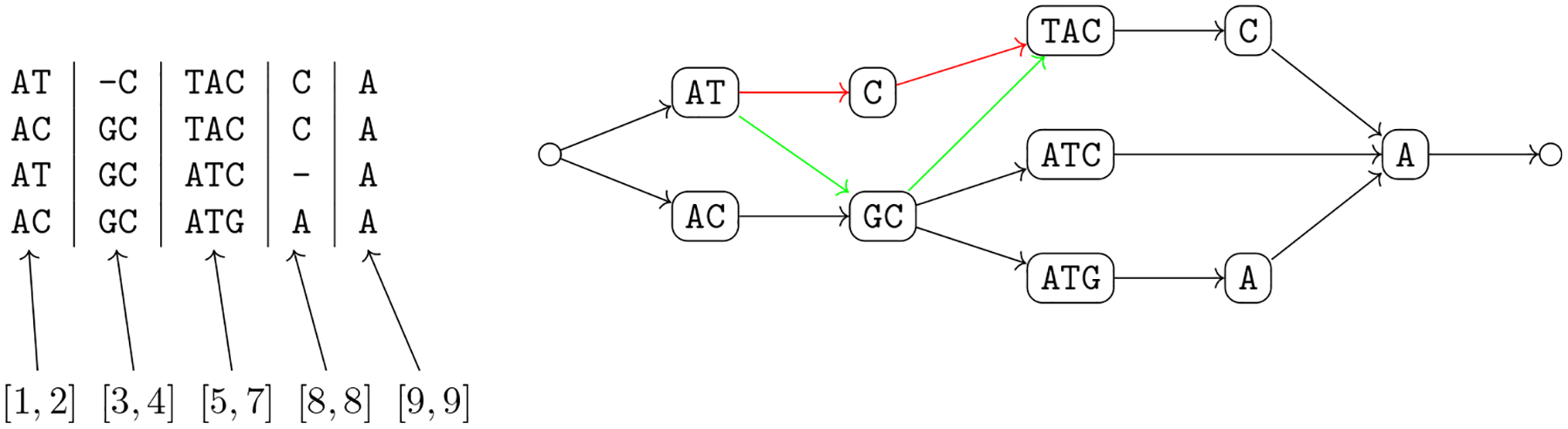
Example of an alignment (left) of four genomes and a corresponding variation graph (right). The set *I* of disjoint intervals is in the lower left part of the figures, and each interval is connected with the corresponding set of columns of the alignment. The variation graph has two dummy vertices: a source and a sink, so that each genome corresponds to source-sink walk in the graph. The alignment of the third genome has a block consisting of only a gap; hence, it does not correspond to any vertex of the graph. The red and the green paths identify a variant, also called bubble, in the graph, since they have the same source and sink, while all other vertices are disjoint

The intuitive idea behind Definition 5 is that we can split the alignment into aligned blocks, where each block that does not consist only of a gap is mapped to a vertex of the variation graph whose label is identical to the block, once all gaps are removed (condition 2a). Moreover, each genome in the alignment corresponds to a walk in the graph (condition 2b), and each arc of the graph corresponds to two consecutive aligned blocks once we discard all aligned blocks consisting only of a gap (condition 2c) in some input aligned sequence. The natural computational problem is then to compute a variation graph compatible with a given alignment (Fig. 3).

**Problem 1** (*graph construction from alignment*) Let $G=\left\{g_{1},\ldots ,g_{m}\right\}$ be a set of *m* aligned genomes, all of length *n*. Then the graph construction from alignment problem asks to find a variation graph *G* that is compatible with G.

The formulation of compatibility in Definition 5 is similar to the formulation of *block graphs* (Ukkonen 2002; Mäkinen et al. 2020), albeit the latter is quite restrictive, e.g., it does not allow cycles.

We note that Problem 1 does not have an objective function that allows to discriminate among all possible graphs that express the genomes in G. Consequently the problem is ill-posed. Moreover, some simple objective functions do not lead to desirable graphs. Given a variation graph *G* = 〈*V*, *A*, *W*〉, we let *W*(*G*) be the set of maximal walks of *G* (i.e., walks starting at a source and ending at a sink of *G*), and note that a walk in *W*(*G*) is not necessarily in *W*. Then a desirable property of a variation graph expressing all genomes in G is that the set of labels of all walks in *W*(*G*) is equal to G. Hence, the objective function that we want to minimize is equal to | {*λ*(*p*) : *p* ∈ *W*(*G*)} |, however, this is trivially minimized by a graph with vertices (and labels) *g*_*i*_ and no arcs. Unfortunately, such a solution means that shared portions among input genomes label different vertices of the graph, while a fundamental motivation of introducing variation graphs is that shared portions should belong to the same vertex. Two possible objective functions that address this shortcoming are to minimize (1) the number of vertices of the graph *G*, or (2) the sum of the length of the labels of *G*. The same trivial graph with vertices (and labels) *g*_*i*_ and no arcs is also the optimum for almost all instances of the first formulation. The second objective function does not discriminate between compacted graphs (whose vertices are labeled by strings) and non-compacted graphs (where all vertices are labeled by a single character), provided that the total length of the labels is the same—instead we would favor a compacted graph, since it is more informative.

The fact that it is hard to find a simple objective function means that, if we desire to find a formal definition of the underlying computational problem, we should explore different directions, such as minimum description length (Grunwald 2004) or multicriteria optimization (Ehrgott 2005) to incorporate different aspects of the desired graph. On the other hand, the literature largely avoids providing a complete formulation of the problem and focuses on the method. For example, consider seqwish (Garrison et al. 2019), which is one of the most widely tools for building a variation graph from an alignment. While the paper contains a very detailed description of the data structures used to represent the resulting graph, almost no mention of the combinatorial properties is present. Clearly, the lack of a formulation of the objective function does not decrease the usefulness of the tool, but it makes harder to benchmark and compare different approach.

Moreover, a multiple alignment is not able to *explicitly* represent certain structural variations, such as inversions or transpositions. For this reason, sometimes we do not have a reliable alignment that can be the building block for constructing a variation graph. In this case, we only start from a set of strings, each representing a genome, and the corresponding computational problem becomes the following to reconstruct the variation graph from the strings.

**Problem 2** (*graph construction from genomes*) Let $G=\left\{g_{1},\ldots ,g_{m}\right\}$ be a set of *m* genomes. Then the graph construction from genomes problem asks to find a variation graph *G* that expresses all genomes in G.

**Fig. 4:**
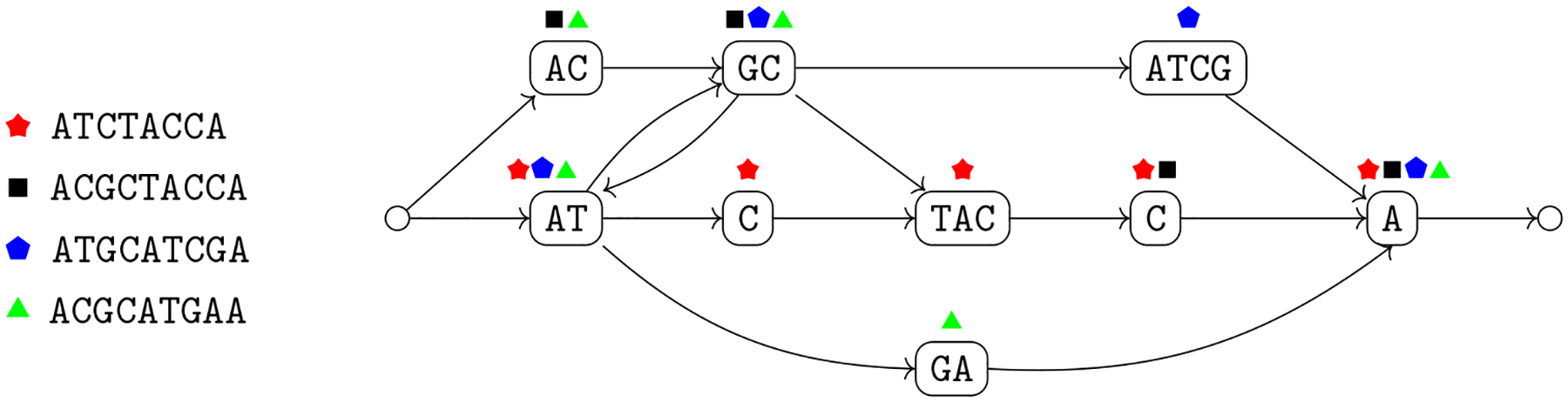
Example of a variation graph constructed from four sequences, each represented by a different colored symbol. We color only vertices to simplify the figure

This new problem is more general than Problem 1, since there is no division into blocks to be respected for all genomes (see Fig. 4 for an example). Moreover, the same argument on the lack of a widely accepted objective function that we have made for constructing the variation graph from an alignment holds also in this case.

For this problem, a simple incremental approach, like the one employed by Minigraph (Li et al. 2020) can be surprisingly effective. In this case, each sequence is aligned against the variation graph (the first sequence is also the initial graph); each portion of the sequence that corresponds to a low quality alignment is a variant that needs to be added to the variation graph. We note that this approach relies heavily on a string-to-graph mapper. The minigraph method incorporates a tailored alignment procedure, inspired by minimap2 (Li 2018), and based on the idea of building (sub)graph chains.

Observe that in minigraph the mapping between genomes and the graph is lost during the construction process. A base-level alignment of the genomes relative to the resulting graph can be obtained by an extension of the Cactus whole genome alignment toolkit (Paten et al. 2011).

## 4 Indexing pangenome graphs

Graphs as large as genome graphs need to be indexed to achieve adequate efficiency for basic operations such as pattern matching or read mapping. Since variation graphs represent walk labels, a simple strategy is to index all relevant walk labels, therefore, mostly reusing the tools that have been developed in text indexing. Most notably, an index can be built to store either *k-mers*, *signatures* or *suffixes* of the walk labels. A *k*-*mer* or *q*-*gram* of a sequence *T* is a substring of length *k* (*q*, respectively) of a sequence *T* and is the building block of de Brujin graphs and of some methods for mapping reads to a genome. In particular, *k*-mer indexing is becoming a popular way of storing huge collections of genomic data (Karasikov et al. 2020). Alternatively, a *signature* or *sketch* of a sequence *T* is a short summary of the sequence given by a vector of numbers that, with high probability, summarizes some *k*-mers of the sequence – see for example MinHash (Berlin et al. 2015). Finally, a suffix sort-based representation of a sequence *T* is given by the self-index structures built upon the notion of Burrows–Wheeler Transform and the FM-index. Generalizing these notions to graphs is a first possible approach to designing pangenome graph representations. The most common approach has been to extend the notion of XBWT (Ferragina et al. 2009) to graphs, first with the GCSA (Sirén et al. 2014; Sirén 2017), which is an index of the prefixes of the strings that can be traversed from each vertex of a directed graph. It has a vertex for each symbol of the sequence, and edges connect symbols that are consecutive in at least one genome sequence (or walk) of the pangenome graph. An alternative approach to indexing is given in (Rakocevic et al. 2019), where pangenome graphs are indexed by using a hash table for *k*-mers extracted from the sequence paths of the graph.

### 4.1 Preliminaries on the BWT

To make this tutorial self-contained, we briefly introduce here the main notions related to the Burrows–Wheeler Transform (BWT). Let *S* be a string that is terminated by a special symbol $ (called *sentinel*). A sentinel appears only at the end of a string and it is smaller than any other symbol of the alphabet Σ. Given a string *S*, its *i*-th character is denoted by *S*[*i*], its substring *S*[*i*]*S*[*i* + 1] ⋯ *S*[*t*] is denoted by *S*[*i* : *t*], and its *suffix* starting at position *i* is denoted by *S*[*i* : ]. Sometimes, instead of the [*i* : *t*] notation, we might use the right-open notation *S*[*i* : *t*) for a substring: in this case the *t*-th character of *S* is not included in the substring, that is *S*[*i* : *t*) = *S*[*i*] ⋯ *S*[*t* – 1].

The *Suffix Array* of *S* (Manber and Myers 1993; Shi 1996) is the array SA s.t. SA[*i*] is equal to *p* if *p* is the starting position in *S* of the suffix of *S* that is the *i*-th suffix of *S* in the lexicographic order of the set of suffixes. The *Longest Common Prefix (*LCP*) array* of *S* is the array LCP s.t. LCP[*i*] is the length of the longest prefix between the (*i* − 1)-th suffix and the *i*-th suffix of *S* in their lexicographic order. Conventionally, LCP[1] = −1.

Given a *n*-long string *S* and the SA of *S*, we denote the *inverse suffix array* as ISA, and define it as ISA[SA[*i*]] = *i* for all *i* = 1, …, *n*. The permutation *ϕ* (Kärkkäinen et al. 2009) is defined as follows: *ϕ*(*i*) = SA[ISA[*i*] – 1] if ISA[*i*] > 1; and *ϕ*(*i*) = SA[*n*] otherwise. In other words, *ϕ*(SA[*j*]) = SA[*j* – 1], for all *j* > 1.

The Burrows–Wheeler Transform (Burrows and Wheeler 1994) of the string *S*, denoted by **BWT**, is a reversible permutation of the characters of *S*. It is the last column of the matrix of the sorted rotations of the text *S*, and can be computed from the suffix array of *S* as **BWT**[*i*] = *S*[*SA*[*i*] – 1], where *S* is considered to be cyclic, *i.e*., *S*[0] = *S*[*n*]. Informally, **BWT**[*i*] is just the symbol of *S* in position *p* − 1 preceding the *i*^*th*^-suffix of *S*. The lexicographic ordering of the suffix starting in position *p* − 1 of *S* is then given by the *LF-mapping*: it is a permutation on [1, *n*] such that SA[**LF**(*i*)] = (SA[*i*] – 1) mod *n*. More precisely, the LF-mapping **LF**(*i*) allows to compute the lexicographic ordering of the suffix of position SA[*i*] − 1 in *S*. Then the LF-mapping allows to virtually traverse the string *S* backwards as explained below using only **BWT**(*S*).

The *backward search* is an operation introduced by Ferragina and Manzini (2005) in order to compute left extension of a given string as follows: given a string *S*, if we know the range **BWT**[*i* : *j*] occupied by characters immediately preceding occurrences of a pattern *P* in *S*, then we can compute the range **BWT**[*i*′ : *j*′] occupied by characters immediately preceding occurrences of *cP* in *S*, for any character *c*. This operation is implemented using: (1) an array *C*[*σ*] that stores the number of symbols in *S* that are smaller than *σ* for each character *σ* and, (2) a (rank) data structure for **BWT**(*S*) that returns how many times a given character occurs up to a specific position of **BWT**(*S*).

Based on the above data structures, a LF-mapping is a last-to-first mapping that associates to a position in the **BWT** a position in the suffix-array and is used by iterations to reconstruct the text from right to left since we are able to compute the preceding symbol of each symbol **BWT**[*i*].

In particular, we can relate function **LF**(*i*) also to character *c* that occurs in **BWT**[*i*] and thus, **LF**(*i*, *c*) is given as the sum *C*[*c*] + **BWT**.*rank*(*i*, *c*), being **BWT**.*rank*(*i*, *c*) the number of *c* symbols occurring in the range **BWT**[1, *i*]. In other words, **LF**(*i*, *c*) gives the position of the specific occurrence of the *c* symbol in the text *S*. Indeed **BWT**(*S*) has the property of preserving the ranking of symbols in *S*. Observe that **BWT**[**LF**(*i*, *c*)] is just the symbol *c*′ preceding *c* in the text *S*, where *c* is in position SA[*i*]. Those functions allow us to quickly solve the pattern matching problem, using only a small space, since the BWT itself can be easily compressed via a run-length encoding and the **BWT**.*rank*() shows increasing values, so we can encode only the difference with the previous value (*i.e*., a delta encoding). In fact, the backward search strategy leads to an *O*(|*P*|) time complexity for counting the number of occurrences of a pattern *P* in a text *S*, given its FM-index. Computing the location of those occurrences is slightly more complex, since it requires a sample of the suffix array of the text, with a time complexity that is very close to that of using a suffix array, that is *O*(|*P*| + *k* log^1+*ϵ*^ |*S*|) where *k* is the number of occurrences of the pattern *P*.

The definition of suffix array has been extended to a set *X* = {*S*_1_, …, *S*_*m*_} of strings by considering the set of the lexicographically sorted suffixes of *X* and by replacing each entry of SA with a pair (*p*, *j*) indicating the length of the suffix (*p*) and the index of the string (*j*) which the suffix belongs to. The *multi-string Burrows Wheeler Transform* (Mantaci et al. 2007) of *X* is the array **BWT** s.t. if *SA*[*i*] = (*p*, *j*), then **BWT**[*i*] is the first symbol of the suffix of *S_j_* starting in position *p*. In other words **BWT** is the concatenation of the symbols preceding the ordered suffixes of *S*.

### 4.2 The positional BWT

The positional BWT (PBWT) is a data structure (Durbin 2014) aiming at representing efficiently a set *X*, or panel, of *m* haplotypes with *n* bi-allelic sites. The notion of PBWT has been generalized to the multi-allelic case (Naseri et al. 2019). From a string-theoretic point of view, the panel *X* is a set of *m n*-long strings over alphabet {0, 1} (for the bi-allelic case) or a generic finite alphabet Σ (for the multi-allelic case). In the following, we introduce the data structure for the multi-allelic case, since it is a straightforward extension of the bi-allelic case. All the results that we discuss have been presented by Durbin (2014) and Naseri et al. (2019). We note that the PBWT has many resemblances with the wavelet matrix proposed by Claude et al. (2015).

The goal of the PBWT is basically to find matches among the haplotypes of *X*, or with respect to an external haplotype and the panel *X*, where a match must involve substrings in the same positions, *i.e.*, two substrings *s*[*i* : *i* + *l*] and *t*[*j* : *j* + *l*] with *i* ≠ *j* are not considered a match even in the case they are equal. To underline this difference, we use the term *haplotype* for an *n*-long string over the (ordered) alphabet Σ with *t* symbols. Let *X* be a set of *m* haplotypes *x*_1_, *x*_2_, …, *x*_*m*_; the positions on each haplotype are indexed from 1 to *n*. Given the haplotype *x*, its *prefix at position k* is its *k*-long prefix *x*[1 : *k*] = *x* [1 : *k* + 1), denoted **pref**(*x*, *k*). The *reversed prefix at position k* is the reverse of **pref**(*x*, *k*), that is the string *x*[*k*] ⋯ *x*[1], and is denoted by **revpref**(*x*, *k*). With a slight abuse of notation, we assume that *x*[*i* : *j*] with *i*>*j* is the empty string. Hence, **pref**(*x*, 0) = **revpref**(*x*, 0) is the empty string. Given two haplotypes, we can define an order for each position.

**Definition 6** (*Position order*) Let *x*_*i*_, *x*_*j*_ be two haplotypes of *X*, and let *k* be an integer not greater than *n*. Then *x*_*i*_ is *smaller than x*_*j*_ *at position k* if and only if:

1. **revpref**(*x*_*i*_, *k*) is lexicographically smaller than **revpref**(*x*_*j*_, *k*), or
2. **revpref**(*x*_*i*_, *k*) = **revpref**(*x*_*j*_, *k*) and *i*<*j*.

Observe that the ordering at position 0 produces the same ordering as the set *X*, that is *x*_1_, …, *x*_*m*_. A *match* between two haplotypes *x*_*i*_ and *x*_*j*_ are two identical substrings *x*_*i*_[*k*_1_ : *k*_2_] and *x*_*j*_[*k*_1_ : *k*_2_] spanning the same position interval [*k*_1_ : *k*_2_]. The match *x*_*i*_[*k*_1_ : *k*_2_] = *x*_*j*_[*k*_1_ : *k*_2_] is *left-maximal* (*right-maximal*, resp.) if it cannot be extended on the left (right, resp.), that is either *k*_1_ = 1 or *x*_*i*_[*k*_1_ – 1] ≠ *x*_*j*_[*k*_1_ – 1] (either *k*_2_ = *n* or *x*_*i*_[*k*_2_ + 1] ≠ *x*_*j*_[*k*_2_ + 1], resp.). We can now define formally the positional BWT.

**Definition 7** (*Positional BWT* (Durbin 2014)) Let *X* = {*x*_1_, ⋯, *x*_*m*_} be a set of *m* haplotypes. The *positional BWT* of *X* is a collection of *n* + 1 pairs of arrays, (*a*_*k*_, *d*_*k*_) for 0 ≤ *k* ≤ *n*, where each *a*_*k*_ is called a *prefix array* and each *d*_*k*_ is called a *divergence array*, defined as follows:

- the prefix array *a*_*k*_ is a permutation of the indexes 1, 2, ⋯, *m* such that *a*_*k*_[*i*] = *j* iff *x*_*j*_ is the *i*-th haplotype of *X* in the ordering at position *k*, *i.e*., considering the *k-*long reverse prefixes,
- the divergence array *d*_*k*_ is such that *d*_*k*_[*i*] is the starting position of the left-maximal match ending at position *k* between the *i*-th and (*i* − 1)-th haplotypes in the ordering at position *k*.

Definition 7 is a departure from the original definition of Durbin (2014) in that the original definition describes the positional BWT as the concatenation of the columns of *X* reordered according to **revprefs**. We argue that the latter is essentially a compact representation of the former, just as the FM-index (Ferragina and Manzini 2005) compactly represents the enhanced suffix array of the text (Abouelhoda et al. 2004). We will conclude this section with an explanation of this fact.

**Fig. 6:**
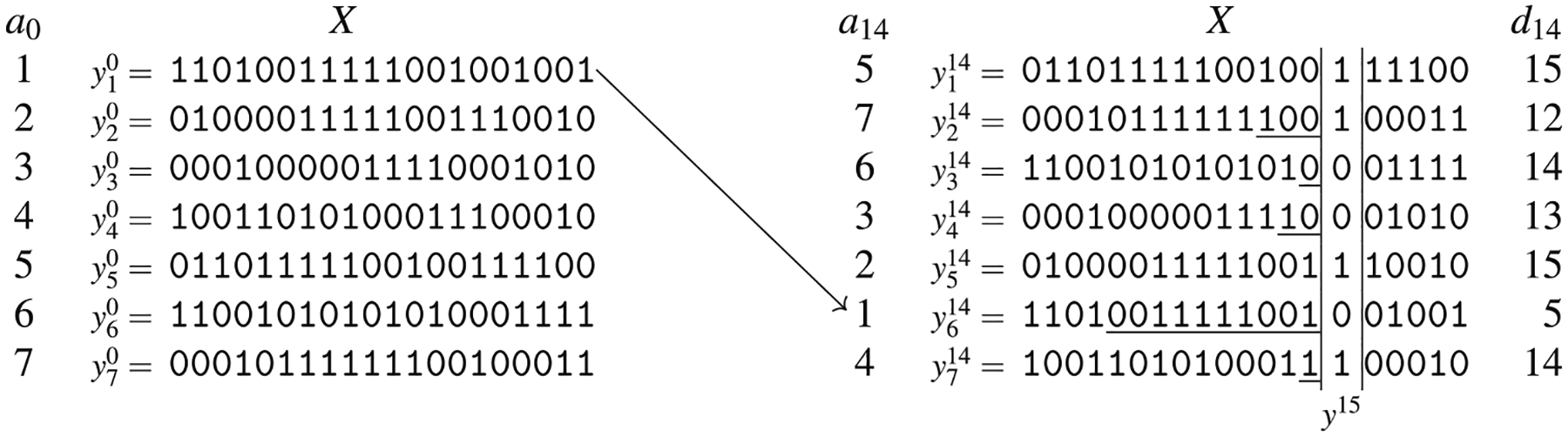
Example of a panel *X* of haplotypes with the original order (left) and with the order induced by *a*_14_ (right). The arrow highlights that *x*_1_ is the 6th haplotype in the order induced by the lexicographic order of the 14-long reverse prefixes (hence, it is denoted with $y_{6}^{14}$). On the right, we reported also the divergence array *d*_14_ and we underlined the left-maximal matches ending at position 14 between each $x_{a_{14}[i-1]}$ and $x_{a_{14}[i]}$. Position 15 is highlighted and the permutation of the symbols (alleles) at that position induced by *a*_14_ is denoted by *y*^15^. That permutation of symbols will be used to compute *a*_15_

For ease of notation, let $y_{i}^{k}$ be $x_{a_{k}\left[i\right]}$. Figure 6 presents an example of the prefix array *a*_14_ and of the divergence array *d*_14_ of a panel *X* of seven haplotypes.

Notice that the Definition 7 means that, for each position *k* and each *i* > 1, there is a left-maximal match between $x_{a_{k}[i-1]}\left[d_{k}[i]:k\right]$ and $x_{a_{k}[i]}\left[d_{k}[i]:k\right]$. Also notice that the prefix array *a*_0_ is the sequence 1, …, *m* since all such prefixes are empty, and *d*_0_ contains only zeroes for the same reason.

If we consider the set of reversed haplotypes, the prefix array *a*_*k*_ is the usual generalized suffix array, restricted to *k*-long suffixes, while the divergence array *d*_*k*_ can be trivially obtained from the LCP array between two consecutive *k*-long suffixes.

Observe that *d*_*k*_[*i*] = *k +* 1 means that no match ending at position *k* exists between haplotypes $y_{i}^{k}$ and $y_{i-1}^{k}$. The following proposition, which is a direct consequence of its definition, is used to compute the divergence array.

**Proposition 1** *Let X be a set of haplotypes and let a*_*k*_*, d*_*k*_ *be the associated prefix and divergence arrays at position k*. *Let i and j be two integers with* 1 ≤ *i* < *j* ≤ *m*. *Then the starting position of the left-maximal match ending at position k of $y_{i}^{k}=x_{a_{k}[i]}$ and $y_{j}^{k}=x_{a_{k}[j]}$ is equal to* max_*i*<*h*≤*j*_{*d*_*k*_[*h*]}.

#### 4.2.1 Computing the prefix and the divergence arrays

The array *a*_*k*_ can be computed from *a*_*k*−1_ with a single scan of all characters at position *k*, with a procedure that is essentially a pass of radix sort.

**Fig. 7:**
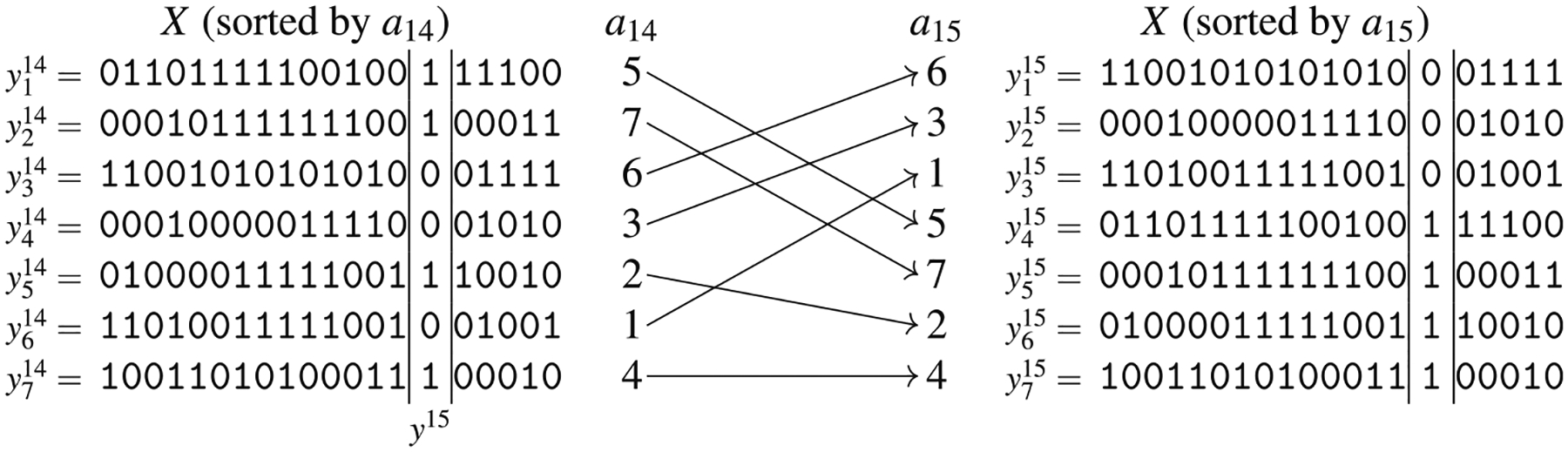
Computing array *a*_15_ from *a*_14_. All the elements of *a*_14_ whose corresponding character in *y*_15_ (*i.e*.,, $x_{a_{k}[\cdot ]}[k]$) is 0 are placed in *a*_15_ before the elements of *a*_14_ whose corresponding character in *y*_15_ is 1

Let *y*^*k*^ be the *m* haplotype characters at position *k* in the order specified by *a*_*k*–1_, that is $y^{k}=\langle y_{1}^{k-1}[k],y_{2}^{k-1}[k],\cdots ,y_{m}^{k-1}[k]\rangle$. Array *a*_*k*_ is computed by sweeping *y*^*k*^ for reordering appropriately the indexes in *a*_*k*−1_. Two observations allow to compute *a*_*k*_ from *a*_*k*−1_: (1) haplotype $y_{i}^{k}$ comes before $y_{j}^{k}$ in the ordering at *k* if $y_{i}^{k}[k]<y_{j}^{k}[k]$ and (2) $y_{i}^{k}$ comes before $y_{j}^{k}$ in the ordering at *k* if $y_{i}^{k}[k]=y_{j}^{k}[k]$ and *i*<*j*. As a consequence, intuitively, in the bi-allelic case we can compute *a*_*k*_ by first placing all the elements of *a*_*k*−1_[*i*] such that $y_{i}^{k}[k]=0$ and then all the elements of *a*_*k*−1_[*i*] such that $y_{i}^{k}[k]=1$ *while keeping the relative order of the elements in each part*. Figure 7 represents this intuition. Clearly, such an idea can be easily extended to the multi-allelic case by considering all the possible symbols.

Also the divergence array *d*_*k*_ can be computed from *d*_*k*−1_ with a single scan of the characters at position *k*.

Let *x*_*p*_ be a haplotype of *X* and let *i* be the index such that *a*_*k*_[*i*] = *p* (hence, $x_{p}=y_{i}^{k}$). Two cases may arise: either (1) $y_{i}^{k}[k]\neq y_{i-1}^{k}[k]$ or (2) $y_{i}^{k}[k]=y_{i-1}^{k}[k]$. In the first case, as the two characters differ, we do not have a non-empty left-maximal match ending at position *k* between $y_{i}^{k}[k]$ and $y_{i-1}^{k}[k]$, thus, *d*_*k*_[*i*] can be conventionally set to *k* + 1. In the second case, there exists a non-empty match ending at position *k* between $y_{i}^{k}[k]$ and $y_{i-1}^{k}[k]$. Let *j* and *j*′ be the indexes such that *a*_*k*−1_[*j*] = *a*_*k*_[*i*] and *a*_*k*−1_[*j*′] = *a*_*k*_[*i* – 1]. Since $y_{i}^{k}[k]=y_{i-1}^{k}[k]=c$, we have that *j*′<*j*. Then, the starting position of the left-maximal match between $y_{i-1}^{k}$ and $y_{i}^{k}$ ending at position *k* (*i.e*., *d*_*k*_[*i*]) is equal to the starting position of the left-maximal match between $y_{j^{\prime }}^{k-1}$ and $y_{j}^{k-1}$ ending at position *k* − 1 which, by Proposition 1, is equal to max_*j*′<*h*≤*j*_{*d*_*k*−1_[*h*]}.

The key observation for obtaining an efficient algorithm is that $y_{j^{\prime }}^{k-1}$ is the most recently seen haplotype with character *c* at position *k*. Hence, while sweeping the characters at position *k*, it suffices to keep, for each allele *σ* ∈ Σ, the running maximum of *d*_*k*−1_ between the current haplotype and the most recently seen haplotype (according to the order induced by *a*_*k*−1_) having *σ* at position *k*. If, at some haplotype $y_{i}^{k}$ we have that $y_{i}^{k}[k]$ is an allele not seen yet, then we must be in case (1) and we set *d*_*k*_[*i*] to *k* + 1. Otherwise we will be in case (2) and we can set *d*_*k*_[*i*] to the running maximum kept for the allele $y_{i}^{k}[k]$.


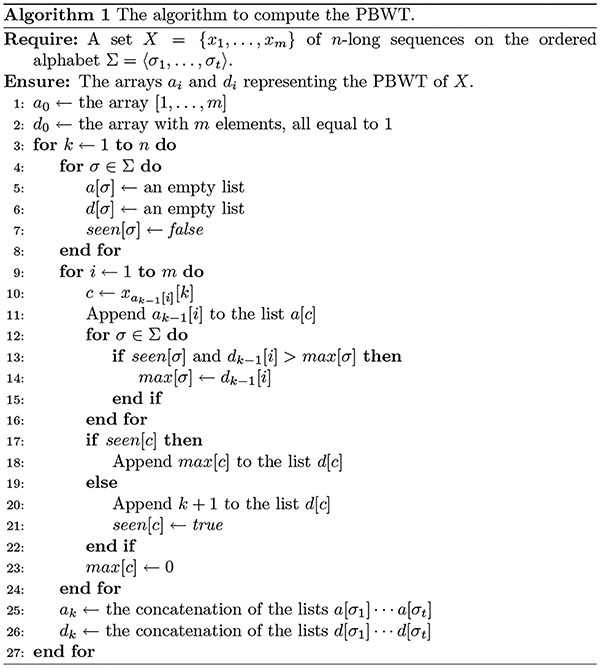


Algorithm 1 formalizes the procedure for computing the entire series of prefix and divergence arrays in a single pass over the panel *X* of *t*-allelic haplotypes. Each iteration *k* of the outer for-loop computes *a*_*k*_ and *d*_*k*_ from *a*_*k*−1_ and *d*_*k*−1_ in *O*(*mt*) time. Hence the total running time is *O*(*nmt*).

**Fig. 8:**
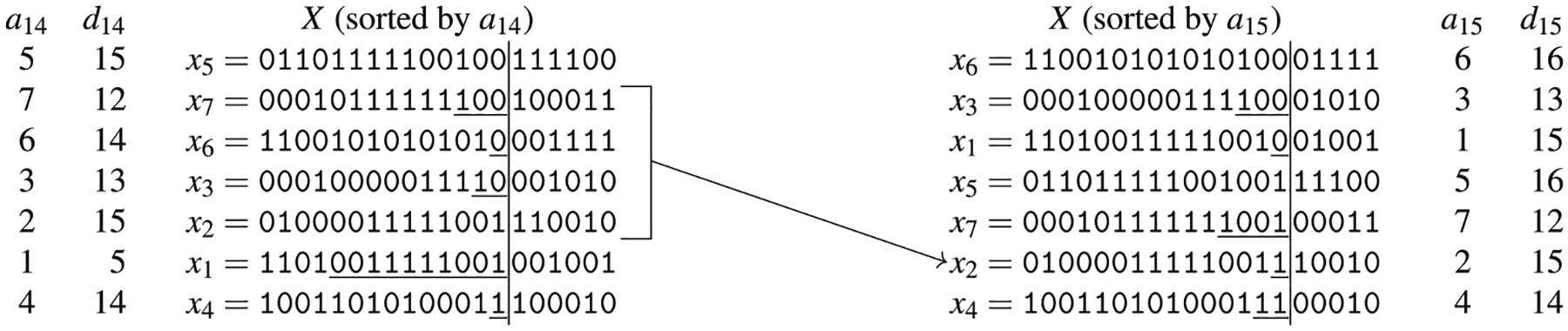
Computing the arrays *a*_15_ and *d*_15_. On the left there are the arrays *a*_14_ and *d*_14_ and the set *X* sorted by the revpref at position 14. On the right there are the set *X* sorted by the revpref at position 15 and the arrays *a*_15_ and *d*_15_. Notice that the set *X* is not sorted explicitly by the algorithm, and is reported here to make easier to understand the algorithm. The interval that is analyzed to compute the value of the divergence array at position 15 associated with *x*_2_ is represented with a square bracket

As an example, we will describe how to compute the arrays *a*_15_ and *d*_15_, given the arrays *a*_14_ and *d*_14_ for the set of haplotypes of Fig. 6. We will use Fig. 8 for illustrative purposes. At the beginning of the scan (lines 9–23), all characters are unseen and the lists *a*[·] and *d*[·] are both empty. The first time we see character 0 (at iteration *i* = 3, corresponding to haplotype *x*_6_) and 1 (at iteration *i* = 1, corresponding to haplotype *x*_5_), the corresponding value of *d*[·] is 15, since the reverse prefix at position 15 and the one that is immediately smaller do not share the character at position 15. For any other haplotype, we check the interval between the most recently seen haplotype that has at position 15 the same character as the current haplotype, and we compute the left-maximal match between those two haplotypes. Consider for example when the current haplotype is *x*_2_ that has the character 1 at position 15. The most recently seen haplotype with the character 1 at position 15 is *x*_7_, and their left-maximal match at position 15 starts at position 15, which is stored in the corresponding entry of *d*_15_. Such position is stored in *max*[1]; the effect of the if at lines 17–23 is that *max*[1] contains the maximum value among all entries of *d*_14_ corresponding to the interval of haplotypes from *x*_7_ (excluded) to *x*_2_ (included) which, by construction of *d*_14_, is exactly the desired starting point.

#### 4.2.2 Maximal matches with at least *L* characters

Using the PBWT we can compute the pairs of haplotypes having a maximal match ending at position *k* with at least *L* characters. Haplotypes between positions *i* and *j* of *a*_*k*−1_, such that all values *d*_*k*−1_[*i* + 1], *d*_*k*−1_[*i +* 2], ⋯, *d*_*k*−1_[*j*] are at most *k* − *L*, share a common (left-maximal) match ending at position *k* − 1 whose length is at least *L*. Such an interval is called an *L*-*block* at position *k*. Observe that only for $y_{p}^{k}$ and $y_{q}^{k}(p,q\in [i,j])$, such that $y_{p}^{k}[k]\neq y_{q}^{k}[k]$, the match ending at *k* − 1 is right-maximal and its starting position can be obtained by performing a range maximum query over the divergence array *d*_*k*_. The algorithm basically separates *d*_*k*−1_ in *L*-blocks and, for each *L*-block the related haplotypes are divided in *t* lists *c*[*σ*] accordingly to their character *σ* at position *k* (i.e., similar to the algorithm for computing the prefix and the divergence arrays). While scanning *d*_*k*−1_, each time a position *i* delimiting the end of a *L*-block is encountered, all the elements of the Cartesian products between all the pairs of lists *c*[*σ*_1_] and *c*[*σ*_2_] (with *σ*_1_ ≠ *σ*_2_) are produced in output. This computation could be performed even in conjunction with the construction of the prefix array *a*_*k*_ and the divergence array *d*_*k*_ – thus avoiding keeping in memory the previously computed arrays *a*_*k*−1_ and *d*_*k*−1_ – using *O*(*m*) in space instead of *O*(*nm*). The running time is bounded by *O*(max(*nmt*; no. of matches)).

#### 4.2.3 Set-maximal matches

**Fig. 9:**
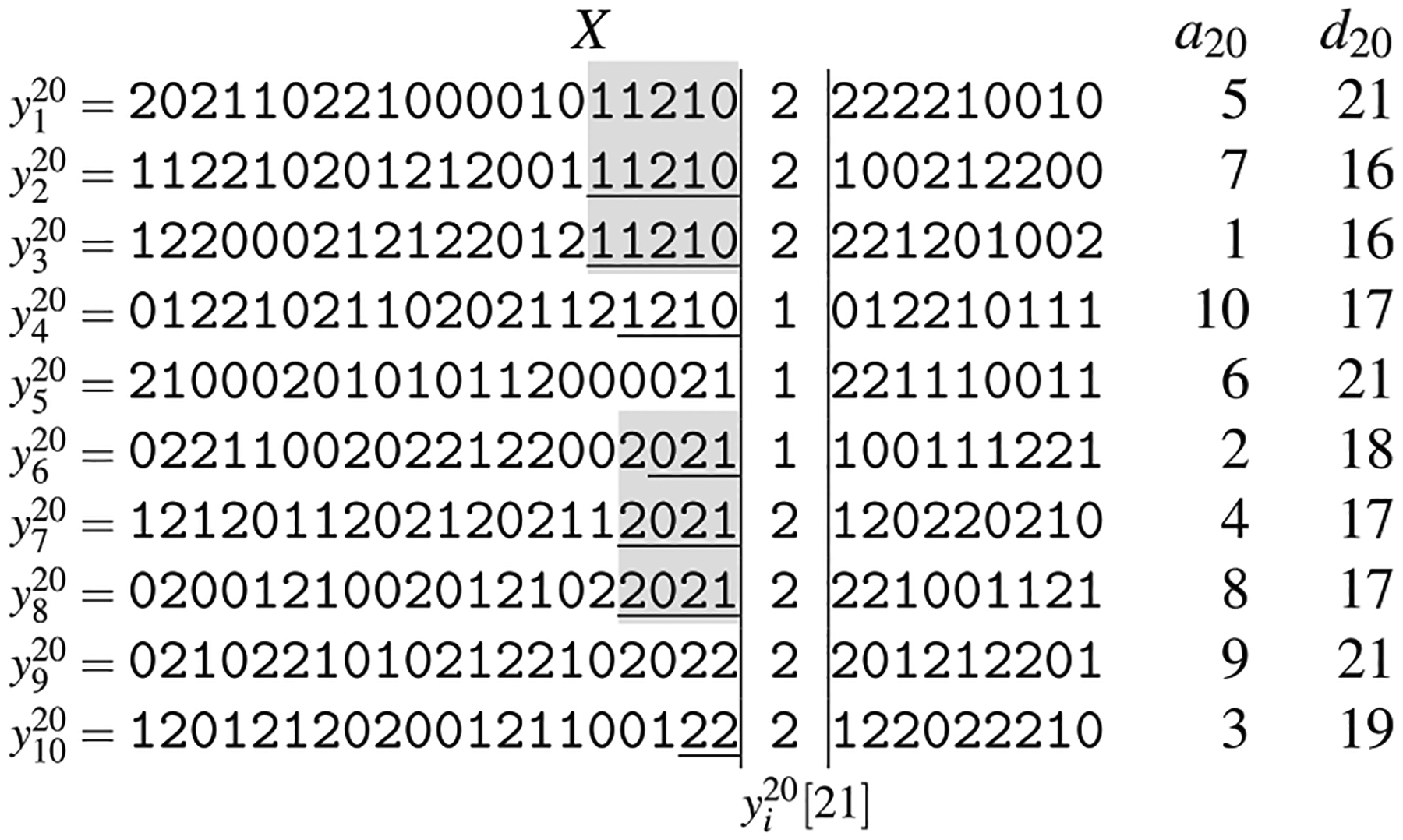
A panel of ten tri-allelic haplotypes in their ordering at 20. Haplotype $y_{2}^{20}$ (which is haplotype *x*_7_ in the original panel *X*) has a candidate set-maximal match from position 16 to position 20 with haplotypes $y_{1}^{20}\left(x_{5}\right)$ and $y_{3}^{20}\left(x_{1}\right)$ since *d*_20_[2] = *d*_20_[3] while *d*_20_[1] and *d*_20_[4] are both greater that *d*_20_[2]. However, since $y_{1}^{20}[21]$ and $y_{3}^{20}[21]$ are both equal to $y_{2}^{20}[21]$, then the match is not right-maximal and, hence, is not set-maximal. It will be found while scanning column 21 or later. Similarly, $y_{6}^{20}$ has a candidate set-maximal match from 17 to 20 with $y_{7}^{20}$ and $y_{8}^{20}$. It is an actual set-maximal match because $y_{6}^{20}[21]$ is different from both $y_{7}^{20}[21]$ and $y_{8}^{20}[21]$. Observe that $y_{7}^{20}$ has not a set-maximal match ending at position 20 because the candidate match from 17 to 20 is with $y_{6}^{20}$ and $y_{8}^{20}$ but $y_{7}^{20}[21]=y_{8}^{20}[21]$ (hence, it will be found while scanning column 21 or later)

A left and right-maximal match *x*_*i*_[*h* : *k*] = *x*_*j*_[*h* : *k*] between haplotypes *x*_*i*_ and *x*_*j*_ such that there is no other haplotype with a match with *x*_*i*_ that properly includes the interval [*h*, *k*], is called a *set-maximal* match of *x*_*i*_ with *x*_*j*_. We note that *x*_*i*_ may have a set-maximal match from *h* to *k* with more than a haplotype in *X*. Observe that haplotype $y_{i}^{k}$ may have a set-maximal match ending at *k* only with the preceding or the following haplotypes in the ordering at *k*. We discuss three cases. The first one is when *d*_*k*_[*i*]<*d*_*k*_[*i* + 1], that is, the left-maximal match between $y_{i}^{k}$ and $y_{i-1}^{k}$ is longer than the left-maximal match between $y_{i}^{k}$ and $y_{i+1}^{k}$. Observe that $y_{i}^{k}$ has a left-maximal match starting at *d*_*k*_[*i*] with all the haplotypes between positions *p* and *i* − 1, where *p* is the smallest position before *i*, such that *d*_*k*_[*j*]≤*d*_*k*_[*i*] for *p*<*j*<*i*. In conclusion, $y_{i}^{k}$ may have a set-maximal match ending at *k* with each haplotype between positions *p* and *i* − 1. Haplotype $y_{i}^{k}$ has actually a set-maximal match with all of these haplotypes if each one of their characters at position *k* + 1 is different from the character at position *k* + 1 of haplotype $y_{i}^{k}$. On the contrary, if even one of those characters is equal to $y_{i}^{k}[k+1]$, then it will be possible to extend the match to the right. Hence, $y_{i}^{k}$ does not have a set-maximal match ending at *k* with such haplotypes. The second case is when *d*_*k*_[*i* + 1]<*d*_*k*_[*i*], that is, the left-maximal match between $y_{i}^{k}$ and $y_{i+1}^{k}$ is longer than the left-maximal match between $y_{i}^{k}$ and $y_{i-1}^{k}$. Again, observe that $y_{i}^{k}$ has a left-maximal match starting at *d*_*k*_[*i* + 1] with all the haplotypes between positions *i* + 1 and *q*, where *q* is the largest position after *i*, such that *d*_*k*_[*j*]≤*d*_*k*_[*i* + 1] for each *i*<*j*≤*q*. In conclusion, $y_{i}^{k}$ may have a set-maximal match ending at *k* with all the haplotypes from position *i* + 1 to position *q*. Haplotype $y_{i}^{k}$ has an actual set-maximal match with all of these haplotypes if each one of their characters at position *k* + 1 is different from the character at position *k* + 1 of haplotype $y_{i}^{k}$. On the contrary, if even one of those characters is equal to $y_{i}^{k}[k+1]$, then it will be possible to extend the match to the right, hence, $y_{i}^{k}$ does not have a set-maximal match ending at *k* with the considered haplotypes. The third case is when *d*_*k*_ [*i*] = *d*_*k*_[*i* + 1]. It is easy to see that this case is the combination of the other two cases, and hence, the set-maximal matches of haplotype $y_{i}^{k}$ ending at position *k* can be found by scanning upwards and downwards in order to find the two position *p* and *q* as described above. Figure 9 represents a panel of haplotypes on which two candidates set-matches have been depicted.

Computing the set-maximal matches is performed while scanning (or computing) the arrays *a*_*k*_ and *d*_*k*_ and checking the characters at position *k* + 1 in the interval [*p*, *i* – 1] or in the interval [*i* + 1, *q*], depending on the values *d*_*k*_[*i*] and *d*_*k*_[*i* + 1]. Since we can stop the upward or downward scan as soon as the check of the following characters fails, the procedure requires *O*(*nmt*) time.

#### 4.2.4 Set-maximal matches between an external haplotype and *X*

The PBWT allows to compute the set-maximal matches of an external haplotype *z* with respect to the panel *X*. Let *e*_*k*_ be the starting position of the longest (left-maximal) match ending at *k* between *z* and some haplotypes of *X* and let *a*_*k*_[*f*_*k*_ : *g*_*k*_) be the portion of *a*_*k*_ related to such haplotypes. While sweeping *z* from left to right, the algorithm computes the values *e*_*k*_, *f*_*k*_ and *g*_*k*_ from the values obtained for *k* − 1. More precisely, it scans the column $y^{k}=\langle y_{1}^{k-1}[k],\cdots ,y_{m}^{k-1}[k]\rangle$ of the *k*-th symbols in the ordering at *k* − 1 and at the same time maintains *c*_*k*_[*σ*], the total number of *σ* ∈ Σ in *y*^*k*^, and *w*_*k*_(*i*, *σ*), the number of characters in the prefix *y*^*k*^[1 : *i*] not greater than *σ* ∈ Σ. Those values allow to compute the interval [*f*_*k*_, *g*_*k*_) of *a*_*k*_ (if it exists) related to the subset of haplotypes in *a*_*k*−1_[*f*_*k*−1_ : *g*_*k*−1_) whose match with *z* starting at *e*_*k*_ can be extended by one position to the right (with character *z*[*k*]). For those familiar with the FM-index, the procedure is similar to the backward search operation. If *f*_*k*_<*g*_*k*_, then there exists some haplotypes (namely, those indicated by *a*_*k*_[*f*_*k*_ : *g*_*k*_ )) such that the match can be extended to position *k* while keeping the starting position at *e*_*k*−1_, hence, we can set *e*_*k*_ = *e*_*k*−1_. Otherwise, if *f*_*k*_ = *g*_*k*_, then no match with haplotypes in *a*_*k*−1_[*f*_*k*−1_ : *g*_*k*−1_) can be further extended. Hence, the haplotypes *a*_*k*−1_[*f*_*k*−1_ : *g*_*k*−1_) have a set-maximal match with *z* from *e*_*k*−1_ to *k* − 1 and such matches are reported. In this case, the algorithm must find the new values *e*_*k*_, *f*_*k*_, and *g*_*k*_ and go on through sweeping *z*. Let *q* be the current value of *f*_*k*_. Since it is possible to prove that *z* is between haplotypes $y_{q-1}^{k}$ and $y_{q}^{k}$ in the ordering at *k*, the algorithm scans the divergence array *d*_*k*_ between those two haplotypes in order to find the left-maximal match with *z* and, in that way, computing the new values *e*_*k*_, *f*_*k*_, and *g*_*k*_.

The running time is *O*(*n*) if we assume that *c*_*k*_[·] and *w*_*k*_(·, ·) have been pre-computed (since they can be used to find the set-maximal matches with different haplotypes external to the panel *X*), while it is *O*(*nmt*) if those values must be computed.

#### 4.2.5 Compact representation of the positional BWT

The first observation that allows to store the panel of haplotypes in a compressed form is that the query algorithms do not directly use the *a*_*k*_[*i*] indexes (that are expensive to store since they are permutations of the range 1…*m*). Indeed, they use the permutation of the symbols in column *k* based on the order of the **revpref** at that position. Similar to the case of BWT (Burrows and Wheeler 1994), such a permutation tends to form long runs of symbols (as those symbols are preceded by similar **revprefs**) that are highly compressible. The information needed to compute the extension of matches (i.e., the rank of the symbols) is similar to those used by the FM-index (Ferragina and Manzini 2005) and thus, can be stored using similar techniques. Using the rank information is also possible to recover the *a*_*k*_ arrays (for reporting purposes) from their sampled representation with negligible impact on performances. Finally, the divergence arrays can be represented as differences between adjacent values. Indeed, adjacent values are similar with high probability, hence, most of the differences should be close to zero and can be represented with fewer bits. In his experiments, Durbin (2014) reports that the GZip-ed storage of the panel requires from ~6 to ~133 times the space required by the PBWT, with the ratio be more favorable as the number of haplotypes increases.

### 4.3 The graph BWT

Observe that the PBWT stores haplotype sequences by encoding which allele each haplotype contains at each position. We can interpret it as a pangenome graph representation restricted to graph topologies where each vertex at position *i* is connected (only) to each vertex at position *i* + 1. The approach was later generalized to arbitrary topologies in the graph extension of the PBWT (Novak et al. 2017). The Graph BWT (GBWT) (Sirén et al. 2020) discussed in this section simplifies the graph extension and makes it more efficient by reducing the problem to indexing strings.

One of the main goals of the GBWT is storing and indexing a variation graph compactly, so that a good locality of reference of the data is maintained. Global information regarding the graph is kept to a minimum, and is usually inferred from local, i.e., vertex-based, information. To achieve this goal, the GBWT stores set of paths, while the variation graph is only inferred from those paths. While the vertices of a genome graph are labeled with a string, the GBWT does not store the labels but only the topology of the graph, where each path is encoded as a sequence of vertex identifiers (Fig. 12).

In other words, each path is a string over the alphabet of vertices, and the graph is a collection of such strings. The GBWT is essentially a multi-string BWT of the collection of strings encoding the paths of the graph. To improve locality of reference, we do not store the BWT as a single string, but as a set of strings **BWT**_*v*_, each corresponding to vertex *v*. The concatenation of all strings **BWT**_*v*_ is the entire BWT. The GBWT inherits the properties of the multi-string BWT. Most notably, given a pattern (i.e., a sequence of vertices) *Q* and the GBWT of a variation graph *G* = (*V*, *E*, *W*), we can answer the following queries:

1. Determine if *Q* is a subpath of at least one path in *W*.
2. Count how many paths in *W* contain *Q* and determine the identifiers of the matching paths.
3. Find the extensions of *Q* that are subpaths of a path in *W*. We may be interested in all maximal extensions in a subgraph, or we may want extend the most promising matches iteratively as long as certain conditions hold.

For each vertex *v*, the GBWT stores the string **BWT**_*v*_ and some additional information to enable fast queries (see Fig. 11).

While the BWT is usually based on sorting the suffixes of the strings and listing the character preceding each suffix in the sorted order, the GBWT works on the reverse strings. It sorts the reverse prefixes of the strings and lists the character following each prefix. Since the strings are the paths of the graph, this allows us to extend a path in the forward direction (that is, according to the path). Consequently, for each vertex *v*, the substring **BWT**_*v*_ corresponds to the prefixes ending with *v*, that is the initial portions terminating in *v* of all paths. Notice the analogy with the fact that each symbol in a regular BWT corresponds to a suffix of the string.

**Definition 8** (*Graph BWT*) Let *G* = (*V*, *E*, *W*) be a variation graph where each walk (path) *W*_*i*_ ∈ *W* is a sequence of vertices 〈*v*_*i*,1_, *v*_*i*,2_, …〉. Then, the *graph BWT* of *G* is the multi-string BWT of the collection of strings $\langle w_{i}=v_{i,1}v_{i,2}\cdots v_{i,|W_{i}|}:W_{i}=\langle v_{i,1}v_{i,2}\cdots v_{i,|W_{i}|}\rangle \in W\rangle$ (under the reverse prefix lexicographic ordering). Moreover, each string **BWT**_*v*_ is the interval of *BWT* corresponding to prefixes of some *w*_*i*_ that end with the vertex *v*.

In the following, we describe the GBWT data structure. Recall that we need to have a compact data structure with a strong locality of reference, which is able to represent a graph version of the LF-mapping of the usual string-based BWT, since the LF-mapping is the main ingredient that is used to answer the queries.

**Fig. 10:**
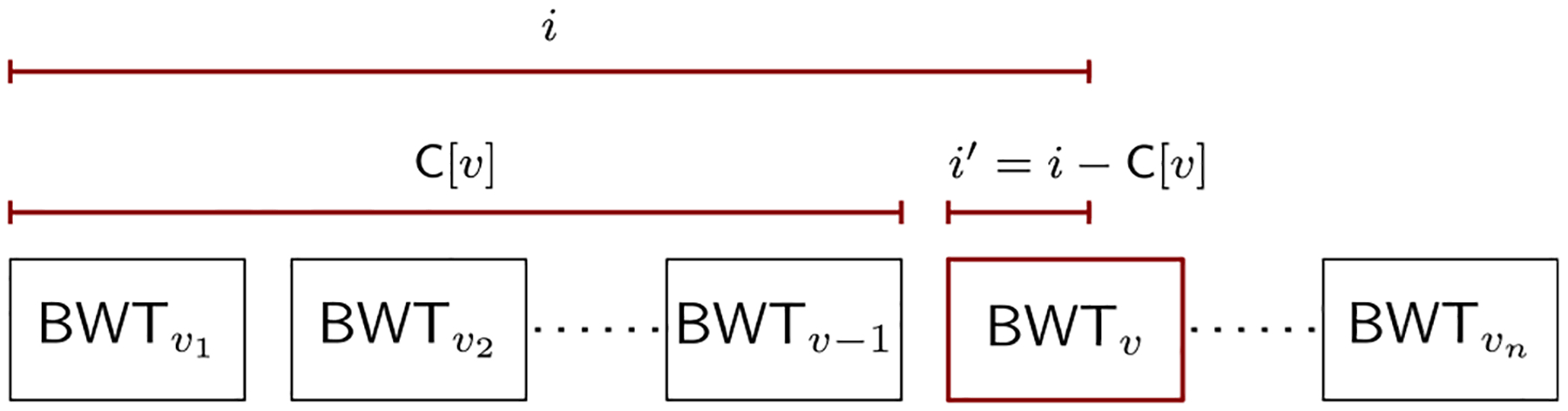
Partitioning the BWT into substrings **BWT**_*v*_ corresponding to vertices *v* ∈ *V* and the representation of BWT offsets *i* as pairs (*v*, *i*′)

Given a graph *G* = (*V*, *E*, *W*), we store the ordered sequence *v*_1_, …, *v*_*n*_ of vertices. We write *v*<*w* if vertex *v* ∈ *V* is before vertex *w* ∈ *V* in the ordering, and use *v* – 1 and *v* + 1 to refer to the predecessor and the successor of *v* in that order. As pangenome graphs typically have an almost linear structure, with |*E*| = *O*(|*V*|), we can use the adjacency list representation for the graph and still obtain, on average, *O*(1)-time access to each outgoing arc. For each vertex *v* ∈ *V*, we store the string **BWT**_*v*_ = **BWT**[C[*v*] + 1 : C[*v* + 1]] that consists of the vertices following *v* in a path of *W* (see Fig. 10). This is based on the same array C as used with the string BWT. For a vertex *v* ∈ *V*, the array stores the overall number of occurrences of all vertices *w* such that *w*<*v* on all paths in *W* as C[*v*].

**Fig. 11:**
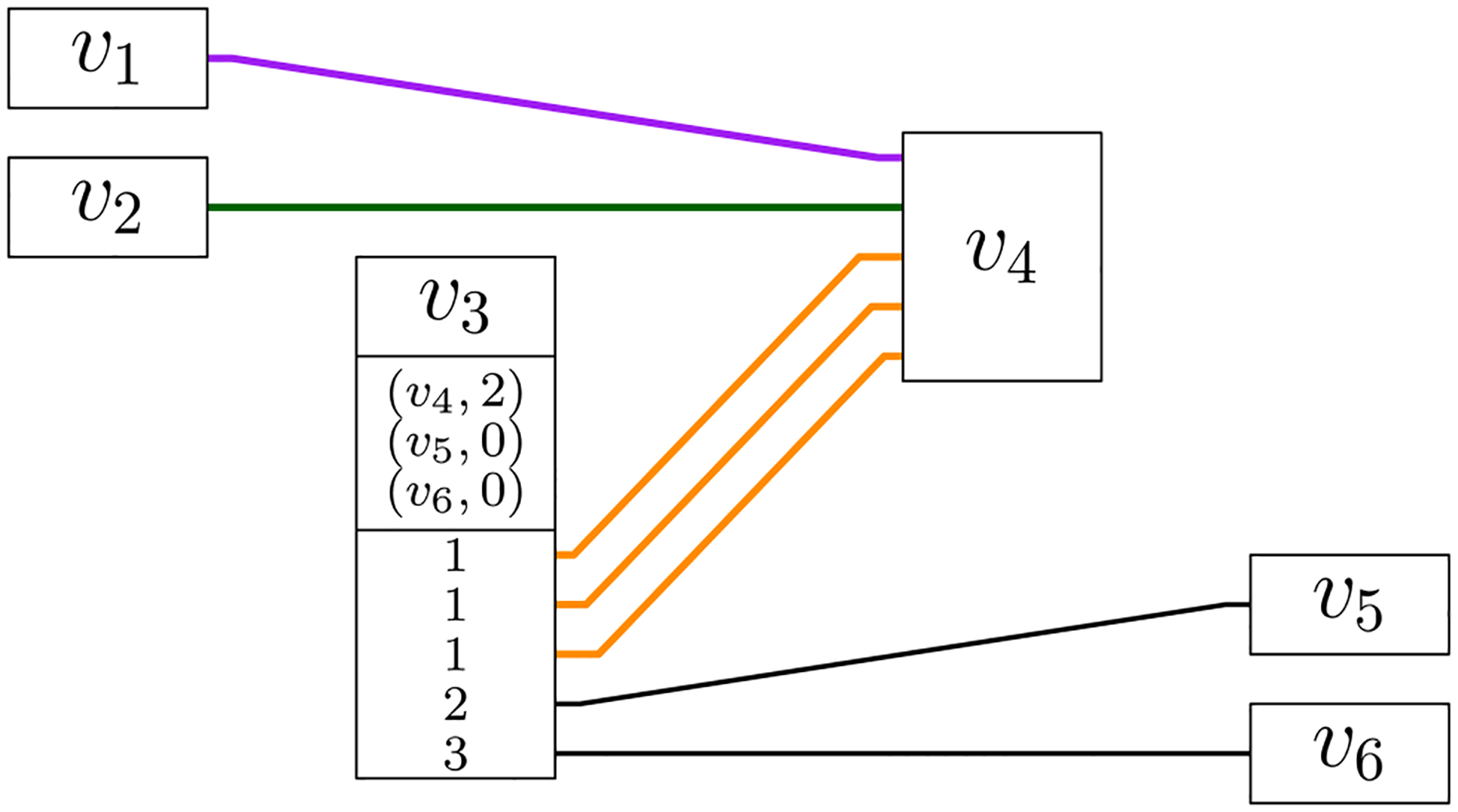
The record for vertex *v*_3_ with outgoing paths to *v*_4_, *v*_5_, and *v*_6_. The top part of the record is the vertex identifier. The middle part stores a pair (*w*, **BWT**.rank(C[*v*], *w*)) for each outgoing edge (*v*, *w*). The bottom part is **BWT**_*v*_ encoded using edge ranks. Observe that there are two paths visiting vertex *v*_4_ from vertices smaller than *v*_3_. Hence, record for vertex *v*_3_ stores the pair (*v*_4_, 2)

The actual data stored for each vertex *v* ∈ *V* is the following:

- The list *N* of vertices *w* such that (*v*, *w*) is an arc of *G*. Notice that this list can be shorter than **BWT**_*v*_ if there are several paths traversing the same arc. For each destination vertex *w*, we also store the number **BWT**.rank(C[*v*], *w*) that is equal to the number of times a path traverses an arc (*v*′, *w*) from a vertex *v*′<*v* (Fig. 11). In the BWT parliance, **BWT**.rank(*i*, *c*) for an integer 1≤*i*≤|**BWT**| and a character *c* denotes the number of occurrences of *c* in the prefix **BWT**[1 : *i*].
- String **BWT**_*v*_ encoding all visits to vertex *v*. For each visit, the string stores the next vertex *w* on the path. The destination vertex is encoded as an arc rank *i* such that *N*[*i*] = *w*. This reduces the space for representing the visits from |**BWT**_*v*_| log |*V*| bits to | **BWT**_*v*_| log *d* bits, where *d* is the outdegree of *v*. Since *d* is constant on the average, a constant number of bits per visit suffices. Additionally, we run-length encode the string **BWT**_*v*_, which can further reduce the space usage if the paths are similar enough (see Sect. 5.2 for a discussion and the definition of run-length encoded BWT).

To avoid storing the array C explicitly, we use (*v*, *i*′) to refer to the BWT offset **BWT**[*i*]. Here *v* is a vertex such that C[*v*]<*i*≤C[*v* + 1] and *i*′ = *i* – C[*v*] the relative offset in **BWT**_*v*_ (see Fig. 10). This simplifies the computation of the values **BWT**.rank(*i*, *w*) that are needed for answering queries. Since *i* = C[*v*] + *i*′, we compute **BWT**.rank(*i*, *w*) as **BWT**.rank(C[*v*], *w*) + **BWT**_*v*_.(*i*′, *w*), where the first term is stored in the record for vertex *v*. The second term, **BWT**_*v*_.rank(*i*′, *w*), is the number of occurrences of *w* in the substring BWT_*v*_ until relative offset *i*′. If the assumptions about the structure of the graph hold, we can compute it efficiently with a linear scan of the compressed **BWT**_*v*_.

The key function for answering queries in a BWT is the LF-mapping **LF**(*i*, *w*) = C[*w*] + **BWT**.rank(*i*, *w*)—see Sect. 4.1. Following our discussion on the substrings **BWT**_*v*_, BWT offsets, and rank queries in the GBWT, we can replace the first term C[*w*] with a reference to vertex *w*. The second term **BWT**.rank(*i*, *w*) is the relative offset in **BWT**_*w*_. It can be computed as **BWT**.rank(C[*v*], *w*)+**BWT**_*v*_.rank(*i*′, *w*), where *i*′ is the relative offset in **BWT**_*v*_. Because all information needed for computing LF-mapping is stored locally in vertex *v*, the memory locality of GBWT queries is better than in ordinary FM-indexes. This is especially true if we store adjacent vertices near each other in memory.

***Example 1*** Consider the record for vertex *v*_3_ in Fig. 11. Let us compute the **LF**-mapping value LF((*v*_3_, 4), *v*_4_). Recall that **LF**(*i*, *c*) is the the number of suffixes smaller than or equal to a hypothetical suffix that starts with *c* and continues with the suffix corresponding to offset *i*. In the GBWT, **LF**((*v*, *i*′), *w*) = (*w*, *j*), where *j* is the number path prefixes ending with *w* that are (in reverse lexicographic order) smaller than or equal to a hypothetical prefix that starts with the prefix corresponding to (*v*, *i*′) and ends with *w*. We compute *j* as the sum of visits to vertex *w* from vertices smaller than *v* and the number of times a path visiting *v* at offset *k*≤*i*′ continues to *w*. The former is stored in the record for vertex *v* and the latter can be computed from **BWT**_*v*_. Since *v*_4_ has 2 visits from vertices with indexes less than *v*_3_ and there are 3 occurrences of *v*_4_ (edge rank 1) in ${\text{BWT}}_{v_{3}}\left[1:4\right]$, we get LF((*v*_3_, 4), *v*_4_) = (*v*_4_, 5).

**Fig. 12:**
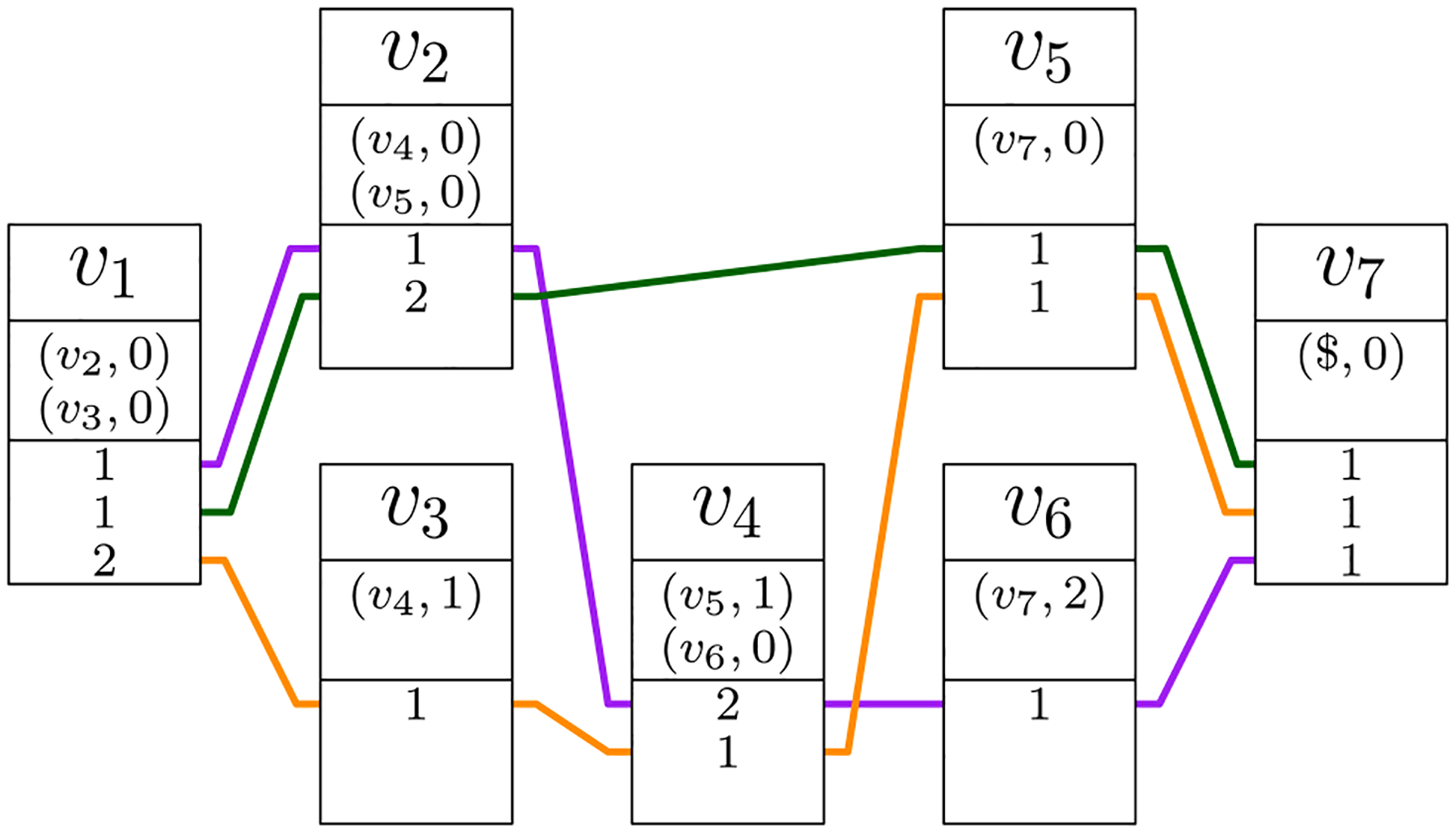
The GBWT in Example 2. As in Fig. 11, the top part of each record is the vertex identifier *v*. The middle part stores a pair (*w*, **BWT**.rank(C[*v*], *w*)) for each outgoing edge (*v*, *w*). The bottom part is BWT_*v*_ encoded using edge ranks

***Example 2*** Figure 12 illustrates the GBWT of the graph induced by three paths *S*_1_, *S*_2_, *S*_3_, one colored purple and consisting of vertices *v*_1_, *v*_2_, *v*_4_, *v*_6_, *v*_7_, one green and consisting of vertices *v*_1_, *v*_2_, *v*_5_, *v*_7_ and finally the orange one consisting of vertices *v*_1_, *v*_3_, *v*_4_, *v*_5_, *v*_7_. The encoded BWT substrings **BWT**_*v*_ for each vertex *v* are:

- *v*_1_ : 112 corresponding to order (*S*_1_, *S*_2_, *S*_3_) of the paths, with the edge of rank 1 to *v*_2_ and edge 2 to *v*_3_;
- *v*_2_ : 12 corresponding to paths (*S*_1_, *S*_2_), with edge 1 to *v*_4_ and 2 to *v*_5_;
- *v*_3_ : 1 corresponding to paths (*S*_3_), with edge 1 to *v*_4_;
- *v*_4_ : 21 corresponding to paths (*S*_1_, *S*_3_), with edge 1 to *v*_5_ and 2 to *v*_6_;
- *v*_5_ : 11 corresponding to paths (*S*_2_, *S*_3_), with edge 1 to *v*_7_;
- *v*_6_ : 1 corresponding to paths (*S*_1_), with edge 1 to *v*_7_; and
- *v*_7_ : 111 corresponding to paths (*S*_2_, *S*_3_, *S*_1_), with edge 1 to nowhere.

***Example 3*** Let us examine another example consisting of paths *S*_1_, *S*_2_, *S*_3_, *S*_4_ where *S*_1_ = *v*_1_, *v*_2_, *v*_4_, *S*_2_ = *v*_1_, *v*_2_, *v*_4_, *S*_3_ = *v*_1_, *v*_2_, *v*_3_, and *S*_4_ = *v*_1_, *v*_3_, *v*_4_. The substrings **BWT**_*v*_ for each vertex are:

- *v*_1_ : 1112 corresponding to paths (*S*_1_, *S*_2_, *S*_3_, *S*_4_), with edge 1 to *v*_2_ and 2 to *v*_3_;
- *v*_2_ : 221 corresponding to paths (*S*_1_, *S*_2_, *S*_3_), with edge 1 to *v*_3_ and 2 to *v*_4_;
- *v*_3_ : 21 corresponding to paths (*S*_4_, *S*_3_), with edge 1 to nowhere and 2 to *v*_4_; and
- *v*_4_ : 111 corresponding to paths (*S*_1_, *S*_2_, *S*_4_), with edge 1 to nowhere.

Another version of the GBWT (Gagie et al. 2017) is a more direct generalization of the positional BWT (Durbin 2014) to graphs. Conceptually, we have a pangenome graph representing some variation using graph topology, with an option to represent rare or less important variants as alternate alleles using another alphabet Σ. The strings are now over alphabet *V* × Σ. Each character (*v*, *c*) represents a visit to vertex *v* ∈ *V* with allele *c* ∈ Σ. Again, we can encode successor vertices with ranks. If *N*[*i*] = *w*, character (*w*, *c*) becomes (*i*, *c*) in the BWT.

***Example 4*** Let us consider now the version that includes the alphabet symbols along the path. We have four paths: *S*_1_ = (*v*_1_, *t*)(*v*_2_, *c*)(*v*_4_, *g*), *S*_2_ = (*v*_1_, *c*)(*v*_2_, *t*)(*v*_4_, *c*), *S*_3_ = (*v*_1_, *g*)(*v*_2_, *c*)(*v*_3_, *g*), and *S*_4_ = (*v*_1_, *c*)(*v*_3_, *t*)(*v*_4_, *c*). In order to use allele symbols in the first real vertex *v*_1_, we start all paths from a special vertex *v*_0_. The BWT is:

- *v*_0_ : (1, *t*)(1, *c*)(1, *g*)(1, *c*) corresponding to paths (*S*_1_, *S*_2_, *S*_3_, *S*_4_), with edge 1 to *v*_1_;
- *v*_1_ : (1, *t*)(2, *t*)(1, *c*)(1, *c*) corresponding to paths (*S*_2_, *S*_4_, *S*_3_, *S*_1_), with edge 1 to v_2_ and edge 2 to *v*_3_;
- *v*_2_ : (1, *g*)(2, *g*)(2, *c*) corresponding to paths (*S*_3_, *S*_1_, *S*_2_), with edge 1 to *v*_3_ and edge 2 to *v*_4_;
- *v*_3_ : (2, *c*)(1, $) corresponding to paths (*S*_4_, *S*_3_), with edge 1 to nowhere and edge 2 to *v*_4_; and
- *v*_4_ : (1, $)(1, $)(1, $) corresponding to paths (*S*_2_, *S*_1_, *S*_4_), with edge 1 to nowhere.

**Fig. 13:**
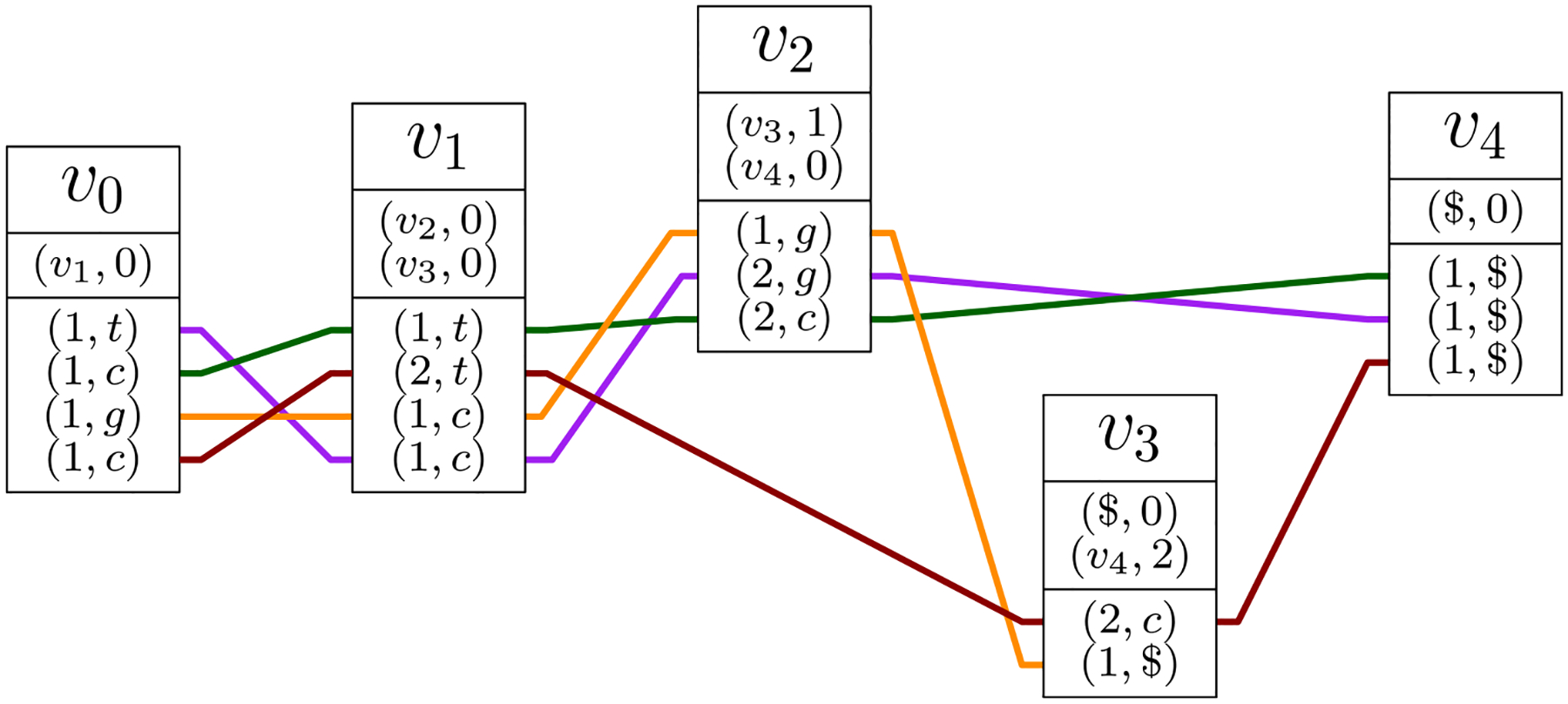
The GBWT from Example 4

See Fig. 13. Note that in this version of the GBWT, the order of path visits in each **BWT**_*w*_ is affected by both the predecessor vertex *v* and the allele symbol *c*.

## 5 Indexing in sub-linear space

Differently from the previous section, we will now discuss a pangenome representation that is not based on graphs, but it relies on the fact that the concatenation *G*_1_ ⋯ *G*_*g*_ of a set of *g* genomes can be viewed as a highly-repetitive string *S*[1 : *n*]—each *G*_*i*_ is a substring of *S* and terminates with a deliminator. The data structure we present, the *r*-index, allows to answer two fundamental queries: counting the number of occurrences in a pattern in *S* (*count*), and locating those occurrences in *S* (*locate*). More complicated queries, such as aligning a sequence read to collection of genomes, can be broken down into count and locate queries. While linear-space indexes—such as the FM-index (see Sect. 4.1)—are well known, they do not fully exploit the repetitive nature of large pangenomes. For example, two terabytes of data would roughly require two terabytes of memory to construct the FM-index. Hence, there has been significant effort in reducing the space requirement of the FM-index while still maintaining the efficiency of performing count and locate queries. In this section, we denote with *P* the query string or pattern to be *P*, and the number of occurrences of *P* in *S* as *occ*.

The main observation is that on large and repetitive data the **BWT** frequently has long equal-character *runs* that could be exploited in order to reduce the size of the construction. We denote *r* as the number of equal-character runs in the **BWT**. Typically, the measure of *n*/*r* describes the extent of repetition in the data and thus, the amount of compression any representation that is dependent only on *r* will obtain—the larger the value, the more compression will likely be obtained. Table 1 illustrates how *n*/*r* varies as the size and number of genomes varies. In a step toward achieving a more efficient construction of the **BWT**, Mäkinen and Navarro (2005) defined the *Run-Length Burrows–Wheeler Transform* (RLBWT).

**Definition 9** Given an input string *S*[1 : *n*], the run-length encoded **BWT** of *S* is the representation RLBWT [1 : *r*] of the **BWT** where each run is represented as the character of the run and its length and where *r* is the number of maximal equal-character runs in the **BWT**, e.g., runs of A’s, C’s and so forth.

The RLBWT can be constructed in a manner that it does not become much slower or larger even for thousands of genomes, which is demonstrated in the following result.

**Theorem 1** (*Mäkinen and Navarro* 2005) *Given an input string S*[1 : *n*], *we can construct its* RLBWT *in O*(*r*)*-space such that we can count the number of occurrences of any pattern P*[1 : *m*] *in O*(*m* log *n*)*-time*.

A compact representation of the RLBWT of the **BWT** of a string *S* consists of a string containing a single character for each run in the RLBWT and a bit vector that marks the beginning of the runs with a 1 (Mäkinen et al. 2010). For example, given the **BWT** = TGCATTAA of the string GATTACAT the RLBWT can be represented with the character string TGCATA and bit vector 11111010. To complete the construction of an FM-index we need also the construction of the suffix array samples in *O*(*r*) space while allowing for efficient queries; this step has remained more elusive. The index of Mäkinen and Navarro can support count queries in *O*(*r*)-space, in order to support locate queries in time proportional to *s*, where *s* is the distance between two samples, they require *O*(*n*/*s*)-space for the SA samples. In practice, these SA samples are orders of magnitude larger in size than the RLBWT. Hence, it was unclear how to sample the SA in a manner that the locate queries were efficient but the sampling of the SA was efficient in practice. More than a decade later, Policriti and Prezza (2017) showed that for a given string *S*[1 : *n*] and a query string *P*[1 : *m*], how to find the interval in the **BWT** containing the *occ* characters preceding occurrences of *P* in *S* in *O*(*m* log log *n*)-time and *O*(*r*)-space. This result, referred to as the Toehold Lemma, demonstrates how to find *one* SA sample in the interval containing a query string *P*. However, it does not fully support locate queries, i.e., locate *all occ* SA samples within that interval. This was solved two years later by Gagie et al. (2020) when they combined the Toehold Lemma, RLBWT of Mäkinen and Navarro (2005), and the definition of *ϕ* to show how to support locate queries in *O*(*r*)-space. In summary, they give the following result.

**Theorem 2** (*Gagie et al*. 2020) *Given an input string S*[1 : *n*], *it is possible to store S in O*(*r*) *space so that we can find all the occ occurrences of any pattern P*[1 : *m*] *in S in O*((*m* + *occ*) log log *n*)*-time*.

The authors refer to the data structure behind this result as the *r-index*. More precisely, the r-index is an evolution of the FM-index and it consists of the RLBWT and a SA sampling that stores the SA values in the positions corresponding to the beginning and the end of every equal-character run in the RLBWT (Gagie et al. 2020). The elucidation of the *r*-index was deemed to be a significant breakthrough as it indicates how the SA can be sampled in space proportional to *r* while allowing for efficient locate queries. However, in some sense it lacked practicality because it was unclear how to efficiently construct it for large genomic databases. Lastly, it it worth nothing that since the introduction of the r-index, other sub sampling approaches have been described and shown to gain improvements in practice (Cobas et al. 2021).

### 5.1 How to construct the r-index

As previously mentioned, Gagie et al. (2020) did not describe how to build the *r*-index – this was shown in a series of papers (Kuhnle et al. 2020; Mun et al. 2020; Boucher et al. 2019). In particular, Boucher et al. (2019) introduced *Prefix Free Parsing (PFP)*, which takes as input a string *S*, window size *w*, and a prime *p* and produces a *dictionary* of substrings of *S* and a *parse* of *S*, that is a sequence of substrings in the alphabet (Kreft and Navarro 2013) – and showed how to build RLBWT from the dictionary and parse. Throughout this section, we denote the dictionary as *D* and the parse as *P*. It was later shown how to build the SA samples in addition to the RLBWT by Kuhnle et al. (2020).

We first describe how to construct the dictionary and parse using PFP. The first step of PFP, is to append and prepend *w* copies of # to *S*, where # is a special symbol that is lexicographically smaller than any element in the alphabet. Hence, given a string *S*, we augment it to contain #^*w*^*S*#^*w*^. We note that this definition is equivalent to the original that considers the circular string *S*#^*w*^. Next, we define the set of trigger strings *T* to consist of the string #^*w*^ and all *w*-length substrings of *S* whose hash is congruent to 0(mod p) — any hash function can be used. The dictionary *D* = {*d*_1_, …, *d*_|*D*|_} is the largest set of all substrings of #^*w*^*S*#^*w*^ such that the following holds for each *d*_*i*_ in *D*: exactly one proper prefix and exactly one proper suffix of *d*_*i*_ are trigger strings, and no other substring of *d*_*i*_ is a trigger string, where a proper prefix or suffix is one that is non-empty. Notice that *D* can be obtained by traversing *S* from right to left, and extracting the list of substrings (called covering substrings) that begin and end with a trigger string and contain no other trigger string. Then, the dictionary *D* is computed by removing duplicated covering substrings and sorting them lexicograpically. Finally, given our dictionary, we determine the parse *P* by replacing each covering string with its rank in the dictionary *D*.

From the dictionary and parse, we can construct some auxiliary data structures in time and space that are linear in the size of *D* and *P*, including the **BWT** of *P* and the SA of *D*. Next, we lexicographically sort the proper suffixes of the substrings in *D* that have length at least *w*, and store their frequency in *S*. For each such suffix α, all the characters preceding occurrences of α in *S* occur together in **BWT**, and the starting position of the interval containing them is the total frequency in *S* of all such suffixes lexicographically smaller than α. It may be that α is preceded by different characters in *S*, because α is a suffix of more than one substring in *D* but then those characters’ order in **BWT** is the same as the order of the phrases containing them in the **BWT** of *P*. These observations lead to the following result.

**Theorem 3** (*Kuhnle et al*. 2020) *Given an input string S*, *we can compute* RLBWT *and* SA *samples in space and time linear in the size of the dictionary and parse constructed from* PFP.

**Fig. 14:**
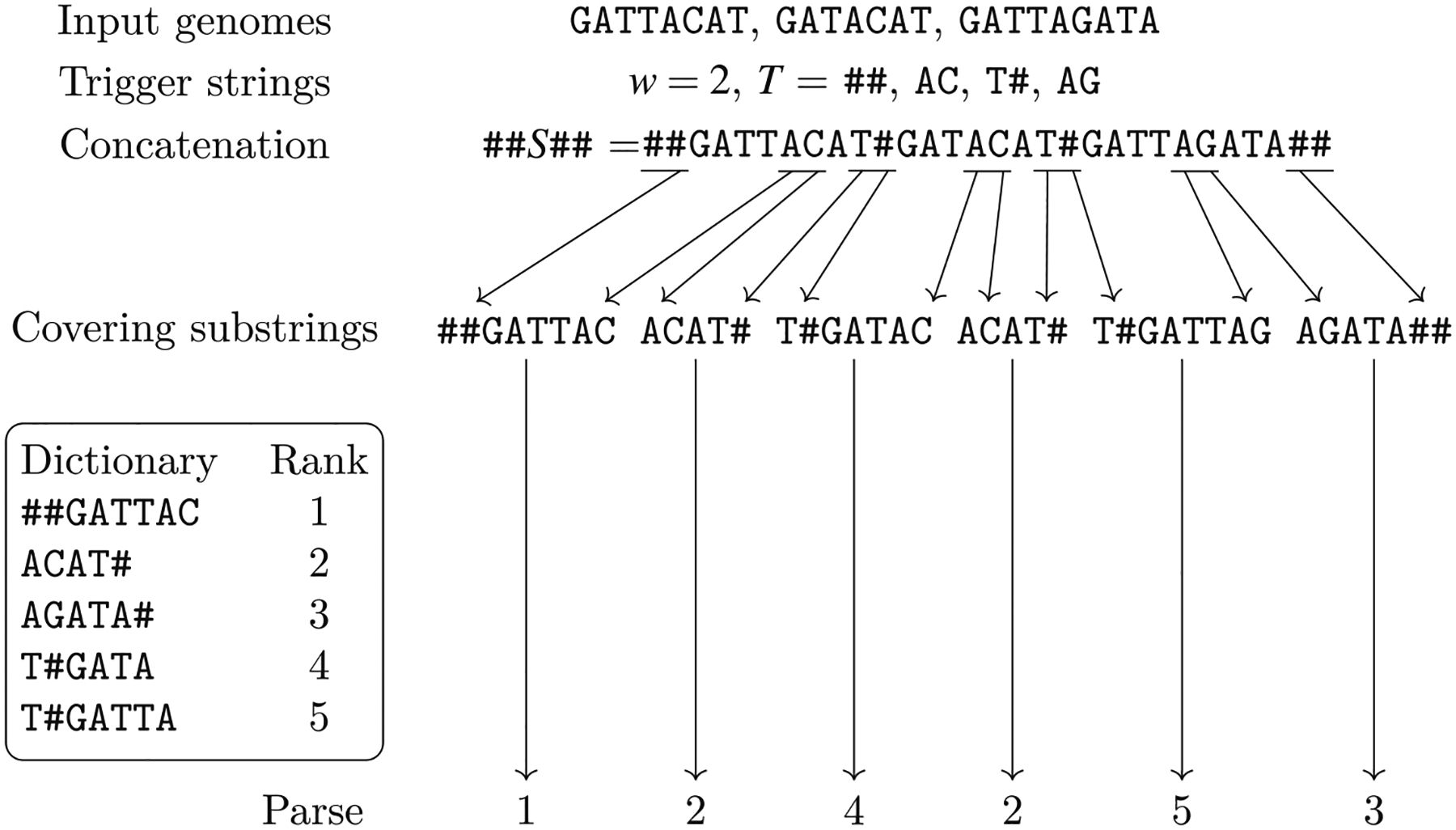
Dictionary and parse of the set GATTACAT, GATACAT, and GATTAGATA of genomes for *w* = 2

Next, we use the example in Fig. 14 to give some intuition as to how to build the suffix array and **BWT** from the dictionary and parse. We remind the reader that suffix array considers all possible rotations of *S* in lexicographical order. These rotations can be obtained using *D* and *P*. To see this, let us consider an expanded form of *D* where we consider all suffixes of *D* that have length greater than *w*, *D*′ = {##GATTAC, #GATTAC, …, TAG}. We can now restate the goal as to how put all sequences of *D*′ in lexicographical order. To see how to accomplish this, we consider all sequences in *D*′ from the first sequences in *D*, ##GATTAC, #GATTAC, GATTAC, ATTAC, TTAC, and TAC, and how to place the second sequence #GATTAC in lexicographical order. To accomplish this we need to consider three cases: (1) if #GATTAC is a prefix of another sequence in *D*′, (2) #GATTAC is a suffix of another sequence in *D*′, or (3) neither is true. Because #GATTAC ends with a trigger strings, it follows that the first case cannot occur. Hence, we only need to consider (2) and (3). If #GATTAC is unique to the first sequence in *D* then it follows that we can place it in lexicographical order without considering *P*. However, if #GATTAC is a suffix of another sequence then *P* can be used to identify the correct lexicographical order. Hence, as the name suggests, that the parse produced by PFP has the property that no suffix of length greater than *w* of any string in *D* is a proper prefix of any other suffix in *D*, which is useful for avoiding the difficult cases in producing the suffix array and **BWT**.

Lastly, we mention that PFP only requires one sequential pass through *S* and thus, can be easily parallelized and performed in external memory. Moreover, it has been recently shown by Boucher et al. (2021) that the products of PFP can be viewed as data structures and be extended to support the following still in *O*(|*P*| + |*D*|)-space: longest common extension (LCE), SA, longest common prefix (LCP) and **BWT**.

### 5.2 How to query the r-index

As previously mentioned, the basic *r*-index can support both count and locate queries but it does not immediately enable finding alignments between query sequences (*e*.*g*., new sequence reads) and reference genomes efficiently. To support these queries, we need to revisit how traditional read aligners use the FM-index (or another index that can perform efficient count and locate queries); after building an index from a small number of reference genomes, majority of them find short exact matches between each read and the reference genome(s), and then extend these to find approximate matches for each entire read. Maximal exact matches (MEMs), which are exact matches between a read *R* and genome *G* that cannot be extended to the left or right, have been shown to be effective seeds for finding full alignments (Li 2013; Miclotte et al. 2016; Vyverman et al. 2015).

**Definition 10** Given a genome *G*[1 : *n*] and a sequence read *R*[1 : *m*], a substring *R*[*i* : *i* + *ℓ* − 1] of length *ℓ* is a *Maximal Exact Match* (MEM) of *R* in *G* if *R*[*i* : *i* + *ℓ* − 1] is also a substring of *G*, but *R*[*i* − 1 : *i* + *ℓ* − 1] and *R*[*i* : *i* + *ℓ*] are not substrings of *G*.

Computing MEMs is equivalent to computing *matching statistics* for *R* (Bannai et al. 2020) which gives, for each position *i* of *R*, the length of the longest substring of *R* starting at position *i* that is also a substring of *G*, and the initial position in *G* of such a substring. We now define formally this notion as follows:

**Definition 11** The matching statistics of *R* with respect to *S* is an array *M*[1 : |*R*|] of (pos, len) pairs such that: (1) *S*[*M*[*i*].pos : *M*[*i*].pos + *M*[*i*].len − 1] = *R*[*i* : *i* + *M*[*i*].len − 1]; and (2) *R*[*i* : *i* + *M*[*i*].len] does not occur in *S*.

**Fig. 15:**
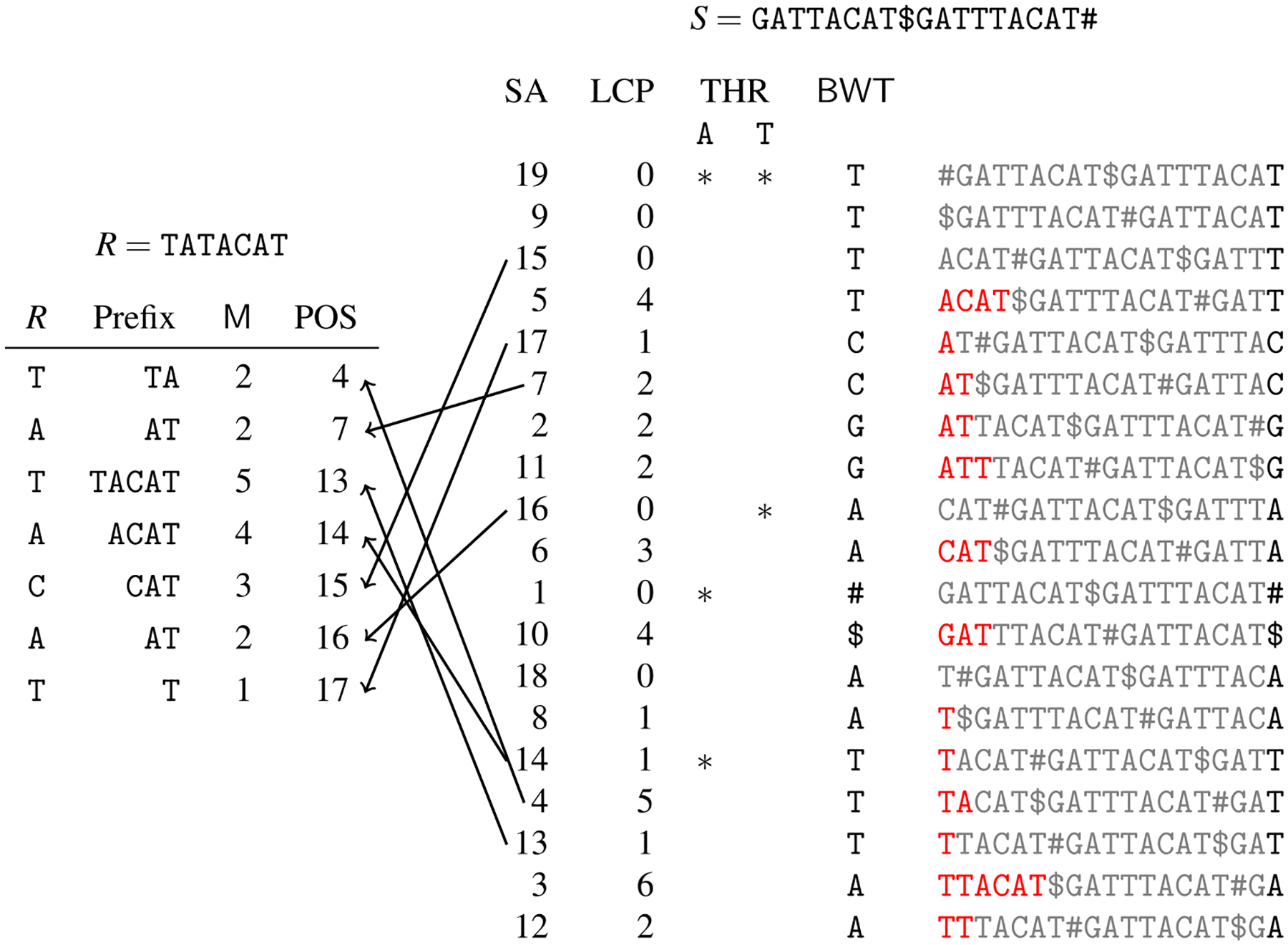
An illustration of the thresholds and matching statistics for identifying pattern *R* (left) in the string *S* (right). We give the longest prefix of the suffix of *R* that occurs in *S*, its length (len), and its position *S* (pos). We give the SA, LCP, the thresholds (THR) and BWT for *S*. The longest common prefix between each consecutive rotations of *S* is highlighted in red

We can compute the matching statistics using a simple two-pass algorithm: first, working right to left, for each suffix of *R* we find the position in *S* of an occurrence of the longest prefix of that suffix that occurs in *S*; then, working left to right, we use random access to *S* to determine the length of those matches. After computing the positions and lengths, you can find the MEMs in a left to right pass of the matching statistics. We note that it is not difficult to see that left to right pass to calculate the lengths and the left to right pass to calculate the MEMs can be done simultaneously. In Fig. 15 we have a query string *R* = TATACAT and *S* = GATTACAT$GATTTACAT#. The position (*POS*) in the suffix array are determined from a right to left pass (which we describe later). For example, we consider the longest common prefix of the suffixes in the following order: T, AT, CAT, …, TATACAT. Considering, ATACAT, which is the second to last suffix considered, we see the longest common prefix of ATACAT that occurs in *S* is AT and one of the occurrences is at position 7 in the suffix array. Next, we can go from left to right to find the lengths and thus, the length of longest match. For example, if we consider ATACAT, we go to *S*[7] and extract all characters until we have a mismatch. On first consideration this may appear to be slow in practice but as Bannai et al. (2020) pointed out, using a compact data structure that supports random access to *S* in *O*(log log *n*)-time, this can be accomplished in *O*(*m* log log *n*)-time and small space. We should note that after finding the position, say *p*, of a single MEM *ϕ* can be used to access the SA from *p* and find all MEMs.

Given the definition of matching statistics, the next question arises as to how to compute them efficiently. A small auxiliary data structure that gives random access to *S* is needed for computing the lengths of the matches. Thus, we need an auxiliary data structure to compute positions—we will clarify why this is needed using our previous example. Given our string *R* = TATACAT, we assume that we have found the position in *S* of the longest prefix of the suffix of ACAT, which is the string itself and occurs in *S* at position 14. We next move to right by one position and attempt at finding the longest match for TACAT, this can be accomplished using the backward search algorithm. This allows us to obtain the position 13 for TACAT. Next, we attempt to extend this match by the rightmost character (A) using backward search and we see that we have a mismatch as ATACAT does not occur in *S* so we are stuck and it is not obvious how to continue computing the matching statistics at the position. Bannai et al. (2020) devised the ingenious concept of *thresholds* that guides the computation of the matching statistics at such points. Collectively, the thresholds is a small data structure that stores a position for each pair of consecutive runs of the same character in the **BWT**, where the position corresponds to the minimum LCP value in the interval between them. For example, in Fig. 15, we see that there exists a threshold at position 16 because it has the smallest LCP value between the run of T’s ending at 17 and the run of T’s starting at 14. If *R*[*i* − 1 : *j*] matches to some position within the range of 17 to 14 but there does not exist a match to T*R*[*i* − 1 : *j*], then we know the longest common prefix with T*R*[*i* − 1 : *j*] is either at the position of the last T of the preceding run of T’s or the first position of the succeeding run of T’s. The thresholds act as a guide to which of these positions it is. If the previous match is a position prior to the threshold then you jump up to the previous run and if it is below the threshold then you jump down to the previous run (Bannai et al. 2020). How to construct efficiently the thresholds with the *r*-index has been later accomplished (Rossi et al. 2021), thanks to an equivalent definition of thresholds (Definition 12), as an addition to PFP.

**Definition 12** Given a text S, let **BWT**[*j*′ : *j*] and **BWT**[*k* : *k*′] be two consecutive runs of the same character in **BWT**. We define a position *j*<*i*≤*k* to be a *threshold* if it corresponds to the minimum value in LCP[*j* + 1 : *k*].

In Fig. 15, we illustrate the thresholds and matching statistics. Revisiting our previous example, we see that the current match of TACAT will occur at position 13 and ATACAT does not occur within *S*. 13 is below the threshold for A (14) so jump down to position 3 and then continue with backward search. Together these concepts summarize how MEM queries can be supported using the *r*-index:

- Construct the r-index with thresholds using the version of PFP of Rossi et al. (2021)
- Given a sequence read *R* calculate the matching statistics of *R* using the thresholds.
- Find the MEMs for *R* using the two-pass algorithm defined above.

Lastly, we note that other exact matches—such as matching *k*-mers—can be used as seeds for alignment and be found nearly identically to that of MEMs in the *r*-index.

## 6 Application scenarios in pangenome graphs

In the following we discuss specific application frameworks.

### 6.1 Haplotype and genotyping in pangenomics and pantrascriptomics

The data structures presented in the tutorial have various application in the analysis of haplotypes and in genotyping variants at population scale level. The Graph Burrows–Wheeler Transform has been recently used by Sirén et al. (2020) to efficiently build a whole-genome index of 5,008 haplotypes of 1KGP (The 1000 Genomes Project Consortium 2015). It is important to note that the GBWT presented by Sirén et al. (2020) is different from the original graph positional BWT proposed by Novak et al. (2017) and leads to a more practical and efficient representation of haplotype-aware indexes, i.e., indexes of pangenome graphs where paths represent the distinct haplotypes in the individuals. These indexes are becoming extremely useful in many applications, since haplotypes are able to distinguish specific SNPs that are relevant in personalized medicine. Sibbesen et al. (2021) used the GBWT to represent a pangenome graph for haplotypes that is annotated with the additional information of a splicing graph. Then quantification of transcripts from RNA-seq data is obtained by taking into account the haplotype information and then by implementing an RNA-seq aligner to the pangenome graph. The alignment of RNA-seq data to splicing graphs is a problem originally considered by Denti et al. (2018). A splicing graph is a graph representing a collection of transcripts and their relation in terms of shared exons. Vertices in the splicing graph are usually exons and edges connect exons that are consecutive in some transcript (Beretta et al. 2014).

The main idea of Sibbesen et al. (2021) is to represent the exons of a splicing graph directly in a pangenome graph by mapping exons to haplotype sequences of the pangenome graph. In this way, they propose a tool for mapping RNA-seq data that is able to take into account haplotype variations in the analysis of transcripts.

### 6.2 Viral haplotype reconstruction

Another application of computational pangenomics arises in viral genome assembly. During infection, viruses replicate their genome billions of times using error-prone replication machinery, hence many of the resulting genomes are inexact copies. These are also referred to as *viral haplotypes*, which together form a viral pangenome. In order to study characteristics such as virulence or drug resistance and to design effective treatments, it is important to identify the different haplotypes present during infection. This can be done through genome sequencing, which produces a collection of short genomic fragments (*reads*) from all haplotypes, combined in a single data set; the goal of viral haplotype reconstruction is to identify all haplotypes present and to estimate the corresponding relative abundances.

**Fig. 16:**
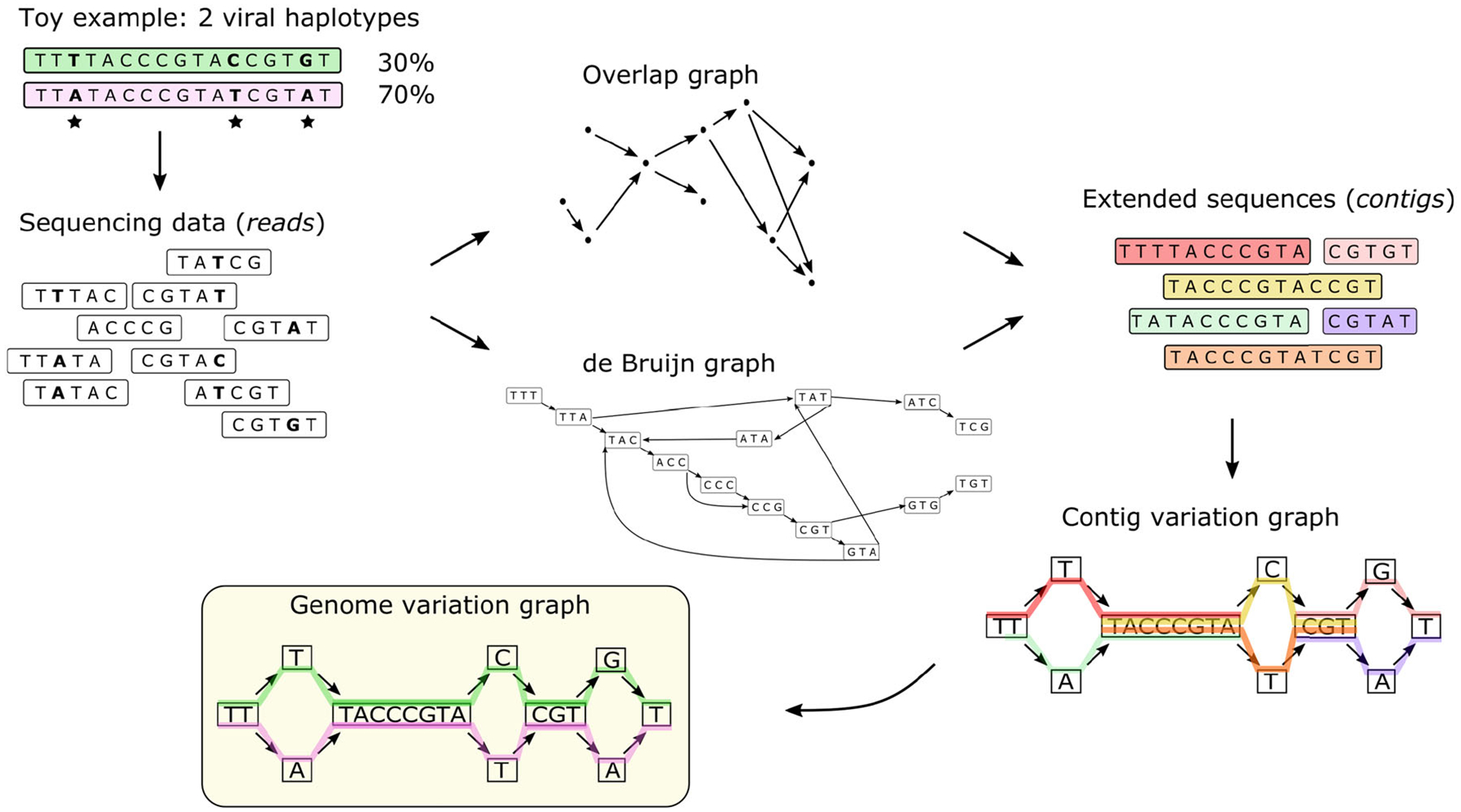
A toy example to illustrate the process of viral haplotype assembly. In this example, the task is to obtain the genome variation graph (a viral pangenome) by reconstructing the viral haplotypes from sequencing data, with haplotypes present at different abundances (here 30 vs. 70%). Stars below the original sequences indicate the three positions where the two haplotypes differ. The three data structures involved in the assembly process are (1) an overlap graph, where vertices represent sequencing reads and arcs indicate suffix-prefix overlaps; (2) a de Bruijn graph, where vertexs represent *k*-mers and arcs indicate overlaps of length *k* − 1; (3) a variation graph, first constructed from the extended sequences (contigs) obtained through genome assembly, which can be transformed into a genome variation graph that represents the full-length haplotypes. *Note that this example is a simplistic representation of reality: sequencing errors are not shown, hence all overlaps between reads are exact*

One of the main challenges in viral haplotype reconstruction is the large amount of reads and the high degree of similarity between those reads. This requires highly efficient graph construction algorithms. Another challenge is to capture the variation within a sample while carefully filtering out any sequencing errors. These challenges are addressed using different types of graphs and benefit greatly from advances in pangenome representations. Below, we describe how different data structures have been used for viral haplotype reconstruction and the advantages and disadvantages of each approach. Figure 16 then presents an instance of a viral sequence data set to illustrate the data structures presented.

#### 6.2.1 Overlap graphs in viral haplotyping

Viral haplotype reconstruction makes use of overlap graphs. Observe that the precise definition of the arcs in an overlap graph can be adjusted to the application: for example, a minimal overlap length threshold *δ* and maximal mismatch rate *ε* can be imposed, meaning that only overlaps of length *L* ≥ *δ* with less than *εL* mismatches give an arc in the overlap graph. In general, complex assembly tasks such as viral haplotype reconstruction require strict arc criteria.

The main idea of approaches that make use of overlap graphs (*e*.*g*., Baaijens et al. 2017; Chen et al. 2018; Töpfer et al. 2014) is that arcs in the graph connect reads originating from the same haplotype. Overlaps between reads are often inexact (*i.e*., Hamming distance > 0) due to sequencing errors. To accommodate such overlaps in the overlap graph, the maximal mismatch rate *ε* should reflect expected sequencing error rates. Furthermore, by choosing a relatively large *δ* one can avoid short overlaps that occur by chance. Finally, base calling quality scores can be used to compute the probability that a pair of overlapping reads originate from the same haplotype; after removing any edges where this probability is below a certain threshold, viral haplotypes can be identified through clique enumeration on the overlap graph (Baaijens et al. 2017; Chen et al. 2018; Töpfer et al. 2014).

The biggest challenge in working with overlap graphs is the graph construction step since the number of potential overlaps is quadratic in the number of input sequences. Naively checking whether a given pair of sequences have any overlap takes *O*(*l*^2^) time, where *l* is the sequence length. Therefore, checking all possible overlaps this way would take *O*(*l*^2^*n*^2^) time, with *n* the number of input sequences. Luckily, there are more efficient algorithms to do this computation. Exact overlaps can be computed efficiently using an FM-index, but this does not work for inexact overlaps. Instead, one can use suffix filters in combination with an FM-index to find approximate overlaps; theoretical runtime remains *O*(*l*^2^*n*^2^) but is much faster in practice (Kucherov and Tsur 2014); Välimälki et al. 2010). This is an exact solution to the approximate suffix prefix overlap problem: it guarantees finding *all* overlaps within specified Hamming distance. Alternatively, heuristic approaches like minimap2 (Li 2018) enable a more efficient, yet approximate, solution to overlap graph construction.

#### 6.2.2 De Bruijn graphs in viral haplotyping

A de Bruijn graph stores the information from the sequencing reads in the form of *k*-mers: each vertex represents a *k*-mer occurring in the reads, and arcs indicate exact suffix-prefix overlaps of length *k* − 1. Such a graph captures shared sequence between haplotypes by collapsing identical *k*-mers and genome assembly is performed by merging simple paths into so-called *unitigs*. De Bruijn graphs are constructed by enumerating and counting all *k*-mers present in the sequencing reads; most algorithms make use of either sorting (*e*.*g*., Kaplinski et al. 2015; Kokot et al. 2017) or hashing (*e*.*g*., Chikhi et al. 2016; Mohamadi et al. 2016) to solve this task efficiently.

In the application of viral haplotype reconstruction, building a de Bruijn graph is very fast because the number of input reads is small compared to mammalian genomes. The main challenge in working with de Bruijn graphs in this setting, is distinguishing sequencing errors from genomic mutations. Standard de Bruijn graph-based assembly algorithms eliminate sequencing errors from the graph by removing low-frequency *k*-mers. This approach is not suitable for viral haplotype reconstruction, because low-frequency *k*-mers can also correspond to low-frequency haplotypes. To avoid this issue, some methods attempt to remove sequencing errors before de Bruijn graph construction by applying error correction software tailored to viral sequencing data (Freire et al. 2020; Malhotra et al. 2016). Alternatively, information on differential coverage (*i.e*., differences in relative abundance between haplotypes) has been used to deconvolute the de Bruijn graph into haplotype assemblies (Fritz et al. 2021).

#### 6.2.3 Variation graphs

Finally, variation graphs are very suitable representations of the genomic diversity found in a viral infection. Given a collection of viral haplotypes, a variation graph can be obtained using the construction techniques discussed earlier. Each viral haplotype can be stored as a path through the graph and relative abundances per haplotype can be added as an additional feature.

In addition to being a suitable representation, variation graphs can also be used as a data structure for haplotype reconstruction. Although algorithms making use of overlap graphs and de Bruijn graphs can assemble haplotype-specific sequences (*contigs*), these are often unable to build complete (*i.e*., full-length) haplotypes: contigs remain shorter than the viral genome. In other words, the assembly techniques described above provide only a partial solution, which can be extended into a full solution using variation graphs (Baaijens et al. 2019, 2020). These algorithms construct a *contig variation graph* from a collection of haplotype-specific contigs, such that the graph organizes the genetic variation that is present in the input contigs. The challenge of constructing this graph is that contigs can have little or no overlap, as they may represent different parts of the genome. Methods that have proven to be useful in this context are VG-msga (Garrison et al. 2018) and poa (Lee et al. 2002), both of which are based on multiple sequence alignment. An alternative approach is to use an all-versus-all aligner like minimap2 (Li 2018) to find all contig overlaps, followed by seqwish (Garrison et al. 2019) for graph construction, but this requires careful filtering of overlaps to obtain a clean graph.

The goal of viral haplotype reconstruction is to find the *genome variation graph* which stores the haplotypes within a viral population, along with an abundance function that gives haplotype abundances. Existing approaches use sequence-to-graph alignment to obtain vertex abundances, from which the haplotypes and their relative abundances are estimated by solving a combinatorial optimization problem on the contig variation graph (Baaijens et al. 2019, 2020). Efficient and reliable variation graph construction is key to algorithms like this.

## 7 Conclusions and open problems

This tutorial on computational pangenomics mainly focuses on presenting the most relevant data structures that are currently used to represent and index pangenomes to facilitate several operations, such as the basic pattern matching and computing matching statistics. After presenting the computational problem of constructing a pangenome graph, we discussed how to face genotyping and haplotyping inference and analysis within a pangenomics framework. The most advanced techniques, namely the positional BWT, the graph BWT, and the r-index have been introduced in the literature recently, and therefore, lead to some important research challenges, while their application to computational pangenomics has been only partially explored. We conclude this tutorial with a discussion on some open problems.

### 7.1 Computing a pangenome graph from overlapping variation graphs

We described the problem of constructing a variation graph in Sect. 3, and most notably as Problem 2, where the instance is a multiple sequence alignment, and we have noticed that the objective function is not always explicit. Devising useful objective functions, adapting the formulation to other instances or desired outcomes are all relevant aspects whose study has already started, for example by considering how to obtain a variation graph from an overlap graph (Eizenga et al. 2021), which is usually considered when assembling a linear genome. This problem is worthy of a deeper investigation, given its relation with the genome assembly problem, as discussed in Sect. 6.2.

An important limitation of current approaches is to avoid complex graph configurations in the output, since those are usually artifacts of the construction procedure, which are removed by manually tweaking some of the parameters of the tool used for building the graph.

A limitation of the formulation that starts from a multiple sequence alignment is that all those sequences have a symmetric role. Instead, it is interesting to exploit the evolutionary history, usually represented by a phylogenetic tree. In this case, we need to refine the objective function to also consider the evolutionary aspects. A possible metaproblem becomes the following.

**Problem 3** (*graph construction from evolutionary related genomes*) Let C a be collection of genome sequences and a scenario of evolutionary events for the genomes. Then the graph construction from evolutionary related genomes asks to find a variation graph *G* that better explains the scenario.

A slightly different approach is based on considering recombination events, which is especially relevant when dealing with a pangenome of haplotypes. In this case, instead of a phylogenetic tree we need to consider a scenario of recombination events, as described by ancestral recombination graphs (Shchur et al. 2019) or by founder graphs (Ukkonen 2002; Mäkinen et al. 2020).

In the following, we give three additional generic open problems, where the specific objective function is not specified, since it depends on the property of the data involved.

**Problem 4** (*graph construction from contigs*) Let C a be collection of partially overlapping sequences (contigs). Then the graph construction from contigs problem asks to find a variation graph *G* that expresses all contigs in C.

We note that this problem is more general than Problem 2 since that problem requires the input sequences appear as source-sink paths in the graph, while they appear as any path in Problem 2. The reason is that we expect the genomes to be highly similar, while contigs can have a small overlap or no overlap at all since they can correspond to different regions in the genome. This means that methods that are based on computing a multiple sequence alignment of contigs are not ideal, since the problem is too hard. In fact, most of the available tools apply a progressive alignment approach. Therefore, the results depend heavily on the order in which the contigs are provided. If the initial alignments regards non-overlapping sequences, then the alignment is not very informative. Moreover, the number of contigs is likely much larger than the number of genomes, making the problem even harder to solve.

**Problem 5** (*graph construction from long reads*) Let R a be collection of long reads. Then, the graph construction from long reads problem asks to find a variation graph *G* that expresses all long reads in R.

This problem is a variant of the problems on contigs or on genomes. Recent sequencing technologies produce reads of 10 to 50 thousand base pairs (Logsdon et al. 2020) but are more error prone compared to short reads or to assembled genomes (or contigs).

Related to these practical problems is the more theoretical problem of building a pangenome graph in sub-linear space. For example, if we consider building and storing a graph using the **BWT**, the question can be sharpened: can we build and store a pangenome graph in *O*(*r*) space and time, where *r* is the number of runs in the **BWT**.

**Problem 6** (*graph construction in sub-linear space*) Let S a be collection of partially overlapping sequences (contigs, genomes or read). Then the sub-linear graph construction problem asks whether you can build a graph *G* that expresses all sequences in S in sub-linear space and time.

### 7.2 Extending the PBWT and the GBWT to missing and erroneous data

The genomes and haplotypes that are indexed by a PBWT or a GBWT are assumed to be complete and error-free, but this is not the case in practice, for multiple reasons including that the raw data contain errors, the tools that manage them are mostly heuristics, and some regions might be absent in the reads. All these prospective issues result in *errors* or *missing* data.

In the best case, errors in a genome or in a haplotype are discovered and corrected; this means that we have to update the PBWT or the GBWT, ideally without computing it from scratch and with a reduced the running time. While there have been some efforts in that direction for the GBWT (Sirén et al. 2020; Eizenga et al. 2020a) that make feasible to update individual genomes in the GBWT, the current state of the art on the PBWT is still lacking. Moreover, it is still unclear what the effect is of a large sequences of operations on the GBWT and on the representations it uses. For example, some problems are (1) to determine if we can build a sequence of operations such that the numbers in the delta encoding explode, (2) if such a sequence can appear in real cases, and (3) to develop a self-balancing procedure that gives some guaranteed sub-linear time complexity for each operation.

Since missing data are fairly common in haplotype panels, it is not surprising that they have already been studied in the context of the positional BWT, where they are represented by a wildcard (Williams and Mumey 2020). A useful notion is that of a haplotype *block*, that is a maximal interval of columns such that (1) a subset of rows of the panel are identical, and (2) it is not possible to extend the interval in any direction. When there are no missing data, blocks can be easily computed using the PBWT. Therefore, an interesting open problem is extending the notion of PBWT to compute matches with missing data. Currently, the complexity of computing blocks with wildcards has asymptotic runtime of *O*(*nm*) for each computed block (Williams and Mumey 2020), with *m* the number of rows and *n* the number of SNP columns of the haplotype panel. An open problem is to compute blocks in a more efficient way, i.e improving the *O*(*nmT*) time complexity, where *T* is the total number of found blocks (Williams and Mumey 2020). Another problem is how to compute approximate blocks (*i.e*., with a small number of mismatches) using the PBWT.

A related problem is to extend the notion of haplotype block to pangenome graphs. In this case, one of the main difficulties is due to the fact that a block consists of portions with the same coordinates, but the notion of coordinates on graphs is not completely established. Moreover, it is interesting to generalize some of the notions discussed in Sect. 5.2 to problems taking as input a graph and a text. For example, defining a proper notion of maximal exact match (MEM) between a sequence read and a graph encoded in the GBWT.

Finally, another problem is the design of a *hierarchical GBWT* that takes the presence of nested structural variants in the pangenome graph into account. Indeed, different genomes may arise from the accumulation of variations. A data structure that allows querying the graph structure at different levels of detail could be useful to represent complex structural variants.

### 7.3 Limitations of pangenome graphs

To provide a balanced point of view on pangenome graphs, we point out some of its current limitations. One type of limitations stems from the fact that stringology has been a wildly successful research field – in particular providing some text indexing techniques (e.g., suffix arrays and the FM-index) that are efficient both in theory and in practice. On the other hand, graph genomes are a recent idea, spurning a research field that is still young. This means that analysis on pangenome graphs becomes orders of magnitude slower than on linear references, and the impact of such analysis needs to be assessed (Chen et al. 2021). Recent research tries to ameliorate this shortcoming by focusing on variant selection approaches that aim to reduce the size of the pangenome graph and speed up mapping (Jain et al. 2021). With the maturation of the field of computational pangenomics, it is expected that tools with better performance will be developed.

Another issue, that is also present in genomics and transcriptomics but is exacerbated in pangenomics, is that a compact representation of several variants can easily result in including spurious variants. In the case of graph genomes, this happens if we naïvely consider all possible paths in a graph. For this reason, variation graphs also store the set of paths corresponding to *true* variants. Still, the construction of such true paths is not trivial, since it requires the use of long reads (Logsdon et al. 2020)—in fact, short reads usually are 100 to 300 base pairs long and only rarely span more than one variant site, while long reads can be 10 to 50 thousand base pairs long. On the other hand, long reads may have a higher rate of sequencing errors than short reads; this may negatively affect the accuracy of read mapping.

A final problem that we want to point out is the extension of pangenomic approaches to more diverse organisms than humans, e.g., a pangenomic approach is also amenable for plants. However, plant genomes present a variability that is much higher than in humans. A recent study on maize sequences showed that 40–50% of genomes is unalignable between pairs of inbred lines (Sun et al. 2018), while a much smaller percentage of human genome cannot be aligned between individuals of different descent (Choudhury et al. 2020b; Sherman et al. 2019). For example, a recent study of African population revealed about 3 million previously undescribed variants (Choudhury et al. 2020b) and Sherman et al. (2019) demonstrated that approximately 10% DNA of an African pangenome built on 910 individuals is not in the current human reference genome.

## Figures and Tables

**Fig. 5:**
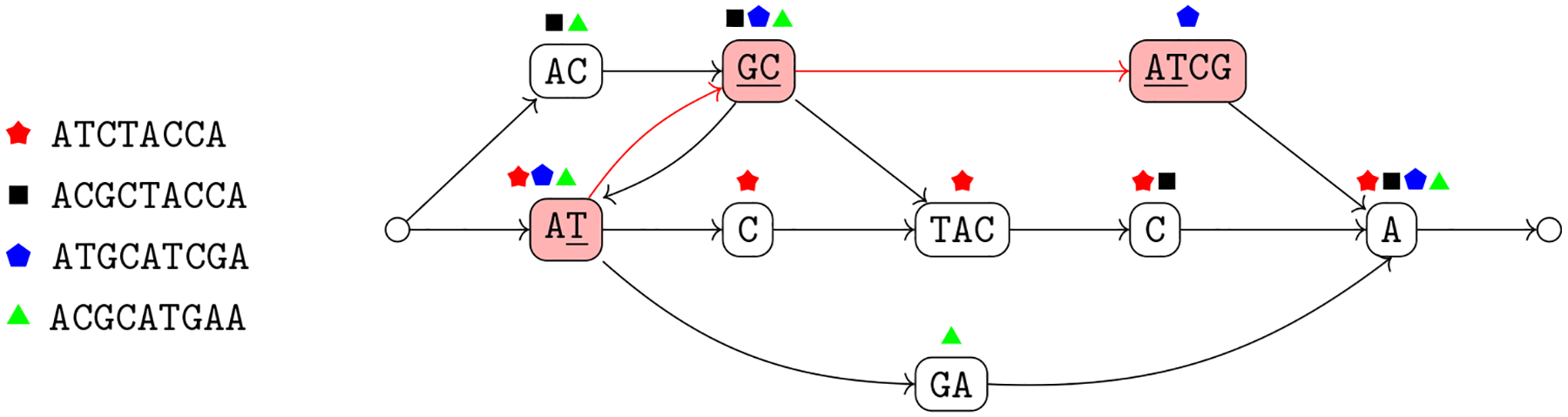
A toy example of how a pattern matches on a variation graph. The pattern is the string TGCAT and the variation graph is the one of Fig. 4. The walk with red vertices and arcs contains the match, but the actual match consists of the underlined portions of the vertex labels. More precisely, the match takes a suffix of the first vertex and a prefix of the last vertex

**Table 1:** Sequence length and *n*/*r* statistic with respect to number of whole genomes for six collections in the 1,000 Genomes Project (1KG) and long-read assembly (LRA) datasets. The table originates from Kuhnle et al. (2020) and is recreated here with permission from the authors

No. of Genomes	Sequence Length (MB)		*n/r*	
	1KG	LRA	1KG	LRA
1	6072	6072	1.86	1.86
2	12,144	12,484	3.70	3.58
3	18,217	17,006	5.38	4.83
4	24,408	22,739	7.13	6.25
5	30,480	28,732	8.87	7.80
6	36,671	34,420	10.63	9.28

## References

[R1] Abouelhoda M, Kurtz S, Ohlebusch E (2004) Replacing suffix trees with enhanced suffix arrays. J Discret Algorithms 2(1):53–86. 10.1016/S1570-8667(03)00065-0

[R2] Baaijens JA, Zine El Aabidine A, Rivals E (2017) De novo assembly of viral quasispecies using overlap graphs. Genome Res 27(5):835–848. 10.1101/gr.215038.116 28396522 PMC5411778

[R3] Baaijens JA, Van der Roest B, Köster J (2019) Full-length de novo viral quasispecies assembly through variation graph construction. Bioinformatics 35(24):5086–5094. 10.1093/bioinformatics/btz443 31147688

[R4] Baaijens JA, Stougie L, Schönhuth A (2020) Strain-aware assembly of genomes from mixed samples using flow variation graphs. bioRxiv:645721. 10.1101/645721

[R5] Ballouz S, Dobin A, Gillis JA (2019) Is it time to change the reference genome? Genome Biol. 10.1186/s13059-019-1774-4 PMC6688217 31399121

[R6] Bannai H, Gagie T (2020) Refining the r-index. Theor Comput Sci 812:96–108. 10.1016/j.tcs.2019.08.005

[R7] Beretta S, Bonizzoni P, Della Vedova G (2014) Modeling alternative splicing variants from RNA-seq data with isoform graphs. J Comput Biol 21(1):16–40. 10.1089/cmb.2013.0112 24200390 PMC3880078

[R8] Berlin K, Koren S, Chin CS (2015) Assembling large genomes with single-molecule sequencing and locality-sensitive hashing. Nat Biotechnol 33(6):623. 10.1038/nbt.3238 26006009

[R9] Bonizzoni P, Dondi R, Klau GW (2016) On the minimum error correction problem for haplotype assembly in diploid and polyploid genomes. J Comput Biol 23(9):718–736. 10.1089/cmb.2015.0220 27280382

[R10] Boucher C, Gagie T, Kuhnle A (2019) Prefix-free parsing for building big BWTs. Algorithms Mol Biol 14(1):13:1–13:15 31149025 10.1186/s13015-019-0148-5 PMC6534911

[R11] Boucher C, Cvacho O, Gagie T, (2021) PFP compressed suffix trees. In: 2021 Proceedings of the Workshop on Algorithm Engineering and Experiments (ALENEX). Society for Industrial and Applied Mathematics, pp 60–72. 10.1137/1.9781611976472.5 PMC8963198 35355938

[R12] Burrows M, Wheeler DJ (1994) A block-sorting lossless data compression algorithm. Tech. rep, Digital Systems Research Center

[R13] Chen J, Zhao Y, Sun Y (2018) De novo haplotype reconstruction in viral quasispecies using paired-end read guided path finding. Bioinformatics 34(17):2927–2935. 10.1093/bioinformatics/bty202 29617936

[R14] Chen NC, Solomon B, Mun T (2021) Reference flow: reducing reference bias using multiple population genomes. Genome Biol 22(1):1–17 33397413 10.1186/s13059-020-02229-3 PMC7780692

[R15] Chikhi R, Limasset A, Medvedev P (2016) Compacting de Bruijn graphs from sequencing data quickly and in low memory. Bioinformatics 32(12):i201–i208. 10.1093/bioinformatics/btw279 27307618 PMC4908363

[R16] Choudhury A, Aron S, Botigué LR (2020) High-depth African genomes inform human migration and health. Nature 586(7831):741–748. 10.1038/s41586-020-2859-7 33116287 PMC7759466

[R17] Choudhury A, Aron S, Botigué LR (2020) High-depth African genomes inform human migration and health. Nature 586(7831):741–748 33116287 10.1038/s41586-020-2859-7 PMC7759466

[R18] Claude F, Navarro G, Ordóñez A (2015) The wavelet matrix: an efficient wavelet tree for large alphabets. Inf Syst 47:15–32. 10.1016/j.is.2014.06.002

[R19] Cobas D, Gagie T, Navarro G (2021) A Fast and Small Subsampled R-Index. In: Proc. of the 32nd Annual Symposium on Combinatorial Pattern Matching, CPM 2021, pp 13:1–13:16

[R20] Compeau PE, Pevzner PA, Tesler G (2011) How to apply de bruijn graphs to genome assembly. Nat Biotechnol 29(11):987–991 22068540 10.1038/nbt.2023 PMC5531759

[R21] Computational Pan-Genomics Consortium (2018) Computational pan-genomics: status, promises and challenges. Brief Bioinform 19(1):118–135. 10.1093/bib/bbw089 27769991 PMC5862344

[R22] Danecek P, Auton A, Abecasis G (2011) The variant call format and VCFtools. Bioinformatics 27(15):2156–2158. 10.1093/bioinformatics/btr330 21653522 PMC3137218

[R23] Denti L, Rizzi R, Beretta S (2018) ASGAL: aligning RNA-Seq data to a splicing graph to detect novel alternative splicing events. BMC Bioinform. 10.1186/s12859-018-2436-3 PMC6247705 30458725

[R24] Denti L, Previtali M, Bernardini G (2019) MALVA: genotyping by mapping-free ALlele detection of known VAriants. iScience 18:20–27. 10.1016/j.isci.2019.07.011 31352182 PMC6664100

[R25] Diestel R (2005) Graph theory. Graduate texts in mathematics, 3rd edn. Springer-Verlag, Heidelberg

[R26] Dilthey A, Cox C, Iqbal Z (2015) Improved genome inference in the MHC using a population reference graph. Nat Genet 47:682–688. 10.1038/ng.3257 25915597 PMC4449272

[R27] Durbin R (2014) Efficient haplotype matching and storage using the Positional Burrows-Wheeler transform (PBWT). Bioinformatics 30(9):1266–1272. 10.1093/bioinformatics/btu014 24413527 PMC3998136

[R28] Ehrgott M (2005) Multicriteria optimization, vol 491. Springer, Berlin. 10.1007/3-540-27659-9

[R29] Eizenga JM, Novak AM, Kobayashi E (2020) Efficient dynamic variation graphs. Bioinformatics 36(21):5139–5144. 10.1093/bioinformatics/btaa640 PMC7850124 33040146

[R30] Eizenga JM, Novak AM, Sibbesen JA (2020) Pangenome graphs. Annu Rev Genomics Hum Genet 21(1):139–162. 10.1146/annurev-genom-120219-080406 32453966 PMC8006571

[R31] Eizenga JM, Lorig-Roach R, Meredith MM, (2021) Walk-preserving transformation of overlapped sequence graphs into blunt sequence graphs with GetBlunted. In: Connecting with Computability - 17th Conference on Computability in Europe, CiE 2021, Proceedings. Springer, LNCS, pp 169–177. 10.1007/978-3-030-80049-9_15

[R32] Ferragina P, Manzini G (2005) Indexing compressed text. J ACM 52(4):552–581. 10.1145/1082036.1082039

[R33] Ferragina P, Luccio F, Manzini G (2009) Compressing and indexing labeled trees, with applications. J ACM 57(1):4:1–4:33. 10.1145/1613676.1613680

[R34] Freire B, Ladra S, Paramá JR (2020) Inference of viral quasispecies with a paired de Bruijn graph. Bioinformatics 37(4):473–481. 10.1093/bioinformatics/btaa782 32926162

[R35] Fritz A, Bremges A, Deng ZL (2021) Haploflow: strain-resolved de novo assembly of viral genomes. Genome Biol. 10.1186/s13059-021-02426-8 PMC8287296 34281604

[R36] Gagie T, Manzini G, Sirén J (2017) Wheeler graphs: a framework for BWT-based data structures. Theoret Comput Sci 698:67–78. 10.1016/j.tcs.2017.06.016 29276331 PMC5727778

[R37] Gagie T, Navarro G, Prezza N (2020) Fully functional suffix trees and optimal text searching in BWT-runs bounded space. J ACM JACM. 10.1145/3375890

[R38] Garrison E (2019) Graphical pangenomics. Thesis, University of Cambridge. 10.17863/CAM.41621, https://www.repository.cam.ac.uk/handle/1810/294516

[R39] Garrison E, Sirén J, Novak A (2018) Variation graph toolkit improves read mapping by representing genetic variation in the reference. Nat Biotechnol 36:875–879. 10.1038/nbt.4227 30125266 PMC6126949

[R40] Garrison E, (2019) seqwish: A variation graph inducer. https://github.com/ekg/seqwish

[R41] Green RE, Krause J, Briggs AW (2010) A draft sequence of the Neandertal Genome. Science 328(5979):710–722. 10.1126/science.1188021 20448178 PMC5100745

[R42] Grunwald P (2004) A tutorial introduction to the minimum description length principle. arXiv:math/0406077 http://arxiv.org/abs/math/0406077

[R43] Guarracino A, Heumos S, Nahnsen S, (2021) ODGI: understanding pangenome graphs. bioRxiv:2021.11.10.467921. 10.1101/2021.11.10.467921 PMC9237687 35552372

[R44] Gusfield D (1997) Algorithms on strings, trees and sequences: computer science and computational biology. Cambridge University Press, Cambridge

[R45] Huang L, Popic V, Batzoglou S (2013) Short read alignment with populations of genomes. Bioinformatics 29(13):i361–i370. 10.1093/bioinformatics/btt215 23813006 PMC3694645

[R46] Jain C, Dilthey A, Misra S, (2019) Accelerating sequence alignment to graphs. bioRxiv:2019.05.27.651638. 10.1101/651638

[R47] Jain C, Tavakoli N, Aluru S (2021) A variant selection framework for genome graphs. Bioinformatics 37(Supplement-1):i460–i467. 10.1093/bioinformatics/btab302 34252945 PMC8336592

[R48] Kaplinski L, Lepamets M, Remm M (2015) GenomeTester4: a toolkit for performing basic set operations - union, intersection and complement on k-mer lists. GigaScience. 10.1186/s13742-015-0097-y PMC4669650 26640690

[R49] Karasikov M, Mustafa H, Danciu D, (2020) Metagraph: Indexing and analysing nucleotide archives at petabase-scale. bioR-xiv:2020.10.01.322164. 10.1101/2020.10.01.322164

[R50] Kärkkäinen J, Manzini G, Puglisi S (2009) Permuted longest-common-prefix array. In: Proc. of the 20th Annual Symposium on Combinatorial Pattern Matching CPM 2009, pp 181–192

[R51] Khorsand P, Denti L (2021) Comparative genome analysis using sample-specific string detection in accurate long reads. Bioinf Adv. 10.1093/bioadv/vbab005 PMC9710709 36700094

[R52] Kokot M, Długosz M, Deorowicz S (2017) KMC 3: counting and manipulating k-mer statistics. Bioinformatics 33(17):2759–2761. 10.1093/bioinformatics/btx304 28472236

[R53] Kreft S, Navarro G (2013) On compressing and indexing repetitive sequences. Theoret Comput Sci 483:115–133. 10.1016/j.tcs.2012.02.006

[R54] Kucherov G, Tsur D (2014) Improved filters for the approximate suffix-prefix overlap problem. In: Moura E, Crochemore M (eds) String processing and information retrieval. Springer International Publishing, Cham, pp 139–148

[R55] Kuhnle A, Mun T, Boucher C (2020) Efficient construction of a complete index for pan-genomics read alignment. J Comput Biol 27(4):500–513. 10.1089/cmb.2019.0309 32181684 PMC7185338

[R56] Lee C, Grasso C, Sharlow MF (2002) Multiple sequence alignment using partial order graphs. Bioinformatics 18(3):452–464. 10.1093/bioinformatics/18.3.452 11934745

[R57] Li H (2013) Aligning sequence reads, clone sequences and assembly contigs with BWA-MEM. arXiv:1303.3997

[R58] Li H (2018) Minimap2: pairwise alignment for nucleotide sequences. Bioinformatics 34(18):3094–3100. 10.1093/bioinformatics/bty191 29750242 PMC6137996

[R59] Li H, Chin J, Durbin R, (2017) GFA: Graphical Fragment Assembly (GFA) Format Specification. http://gfa-spec.github.io/GFA-spec/

[R60] Li H, Feng X, Chu C (2020) The design and construction of reference pangenome graphs with minigraph. Genome Biol. 10.1186/s13059-020-02168-z PMC7568353 33066802

[R61] Logsdon GA, Vollger MR, Eichler EE (2020) Long-read human genome sequencing and its applications. Nature Reviews Genetics 1–18 10.1038/s41576-020-0236-x PMC7877196 32504078

[R62] Magi A, D’Aurizio R, Palombo F (2015) Characterization and identification of hidden rare variants in the human genome. BMC Genomics. 10.1186/s12864-015-1481-9 PMC4416239 25903059

[R63] Mäkinen V, Navarro G (2005) Succinct suffix arrays based on run-length encoding. Nordic J Comput 12(1):40–66

[R64] Mäkinen V, Cazaux B, Equi M, (2020) Linear time construction of indexable founder block graphs. arXiv:2005.09342

[R65] Malhotra R, Wu MMS, Rodrigo A, (2016) Maximum likelihood de novo reconstruction of viral populations using paired end sequencing data. arXiv:1502.04239 10.1109/TCBB.2024.3374595 38451771

[R66] Manber U, Myers G (1993) Suffix arrays: a new method for on-line string searches. SIAM J Comput 22(5):935–948

[R67] Mantaci S, Restivo A, Rosone G (2007) An extension of the Burrows-Wheeler Transform. Theoret Comput Sci 387(3):298–312. 10.1016/j.tcs.2007.07.014

[R68] Miclotte G, Heydari M, Demeester P (2016) Jabba: hybrid error correction for long sequencing reads. Algorithms Mol Biol 11:10. 10.1186/s13015-016-0075-7 PMC4855726 27148393

[R69] Mohamadi H, Chu J, Vandervalk BP (2016) ntHash: recursive nucleotide hashing. Bioinformatics 32(22):3492–3494. 10.1093/bioinformatics/btw397 27423894 PMC5181554

[R70] Mun T, Kuhnle A, Boucher C (2020) Matching reads to many genomes with the r-index. J Comput Biol 27(4):514–518. 10.1089/cmb.2019.0316 32181686 PMC7185317

[R71] Myers E (2005) The fragment assembly string graph. Bioinformatics 21(Suppl. 2):ii79–ii85. 10.1093/bioinformatics/bti1114 16204131

[R72] Mäkinen V, Navarro G, Sirén J (2010) Storage and retrieval of highly repetitive sequence collections. J Comput Biol 17(3):281–308. 10.1089/cmb.2009.0169 20377446

[R73] Naseri A, Zhi D, Zhang S (2019) Multi-allelic positional Burrows-Wheeler transform. BMC Bioinform. 10.1186/s12859-019-2821-6 PMC6551244 31167638

[R74] Novak A, Garrison E, Paten B (2017) A graph extension of the positional Burrows-Wheeler transform and its applications. Algorithms Mol Biol 12:18. 10.1186/s13015-017-0109-9 28702075 PMC5505026

[R75] Paten B, Earl D, Nguyen N (2011) Cactus: algorithms for genome multiple sequence alignment. Genome Res 21(9):1512–1528. 10.1101/gr.123356.111 21665927 PMC3166836

[R76] Paten B, Novak A, Eizenga J (2017) Genome graphs and the evolution of genome inference. Genome Res 27(5):665–676. 10.1101/gr.214155.116 28360232 PMC5411762

[R77] Policriti A, Prezza N (2017) LZ77 computation based on the run-length encoded BWT. Algorithmica 80(7):1986–2011. 10.1007/s00453-017-0327-z

[R78] Popejoy AB, Fullerton SM (2016) Genomics is failing on diversity. Nature 538(7624):161–164. 10.1038/538161a 27734877 PMC5089703

[R79] Rakocevic G, Semenyuk V, Lee WP (2019) Fast and accurate genomic analyses using genome graphs. Nat Genet 51(2):354–362. 10.1038/s41588-018-0316-4 30643257

[R80] Rautiainen M, Mäkinen V, Marschall T (2019) Bit-parallel sequence-to-graph alignment. Bioinformatics 35(19):3599–3607. 10.1093/bioinformatics/btz162 30851095 PMC6761980

[R81] Rizzi R, Beretta S, Patterson M (2019) Overlap graphs and de Bruijn graphs: data structures for de novo genome assembly in the big data era. Quantit Biol 7:278–292. 10.1007/s40484-019-0181-x

[R82] Rossi M, Oliva M, Langmead B, (2021) MONI: A pangenomics index for finding MEMs. In: Proc. of the 25th Annual International Conference on Research in Computational Molecular Biology, RECOMB 2021

[R83] Schneider VA, Graves-Lindsay T, Howe K (2017) Evaluation of grch38 and de novo haploid genome assemblies demonstrates the enduring quality of the reference assembly. Genome Res 27(5):849–864 28396521 10.1101/gr.213611.116 PMC5411779

[R84] Shchur V, Ziganurova L, Durbin R (2019) Fast and scalable genome-wide inference of local tree topologies from large number of haplotypes based on tree consistent PBWT data structure. bioRxiv:2019.02.06.542035. 10.1101/542035

[R85] Sherman RM, Forman J, Antonescu V (2019) Assembly of a pangenome from deep sequencing of 910 humans of african descent. Nat Genet 51(1):30–35 30455414 10.1038/s41588-018-0273-y PMC6309586

[R86] Shi F (1996) Suffix arrays for multiple strings: a method for on-line multiple string searches. In: Concurrency and Parallelism, Programming, Networking, and Security, LNCS, vol 1179. Springer, pp 11–22. 10.1007/BFb0027775

[R87] Sibbesen JA, Maretty L (2018) Accurate genotyping across variant classes and lengths using variant graphs. Nat Genetic 50(7):1054–1059. 10.1038/s41588-018-0145-5 29915429

[R88] Sibbesen JA, Eizenga JM, Novak AM, (2021) Haplotype-aware pantranscriptome analyses using spliced pangenome graphs. bioRxiv:2021.03.26.437240. 10.1101/2021.03.26.437240 36646895

[R89] Sirén J (2017) Indexing variation graphs. In: 2017 Proceedings of the Meeting on Algorithm Engineering and Experiments (ALENEX). Proceedings, SIAM, pp 13–27. 10.1137/1.9781611974768.2

[R90] Sirén J, Monlong J, Chang X, (2021) Genotyping common, large structural variations in 5,202 genomes using pangenomes, the Giraffe mapper, and the vg toolkit. bioRxiv:2020.12.04.412486. 10.1101/2020.12.04.412486

[R91] Sirén J, Välimäki N, Mäkinen V (2014) Indexing graphs for path queries with applications in genome research. IEEE/ACM Trans Comput Biol Bioinf 11(2):375–388. 10.1109/TCBB.2013.2297101 26355784

[R92] Sirén J, Garrison E, Novak AM (2020) Haplotype-aware graph indexes. Bioinformatics 36(2):400–407. 10.1093/bioinformatics/btz575 31406990 PMC7223266

[R93] Stark Z, Dolman L, Manolio TA (2019) Integrating genomics into healthcare: a global responsibility. Am J Human Genetics 104(1):13–20 10.1016/j.ajhg.2018.11.014 PMC6323624 30609404

[R94] Sun S, Zhou Y, Chen J (2018) Extensive intraspecific gene order and gene structural variations between Mo17 and other maize genomes. Nat Genet 50(9):1289–1295. 10.1038/s41588-018-0182-0 30061735

[R95] Tettelin H (2005) Genome analysis of multiple pathogenic isolates of streptococcus agalactiae: implications for the microbial “pan-genome”. Proc Natl Acad Sci 102(39):13950–13955. 10.1073/pnas.0506758102 16172379 PMC1216834

[R96] The 1000 Genomes Project Consortium (2015) A global reference for human genetic variation. Nature 526(7571):68–74. 10.1038/nature15393 26432245 PMC4750478

[R97] Töpfer A, Marschall T, Bull R (2014) Viral quasispecies assembly via maximal clique enumeration. PLoS Comput Biol 10(3):e1003,515. 10.1371/journal.pcbi.1003515 PMC3967922 24675810

[R98] Ukkonen E (2002) Finding founder sequences from a set of recombinants. In: Algorithms in Bioinformatics, WABI 2002. Springer, pp 277–286. 10.1007/3-540-45784-4_21

[R99] Välimälki N, Ladra S, Mälkinen V (2010) Approximate all-pairs suffix/prefix overlaps. In: Combinatorial Pattern Matching, CPM 2010, LNCS, vol 6129. Springer, pp 76–87. 10.1007/978-3-642-13509-5_8

[R100] Vyverman M, De Baets B, Fack V (2015) A long fragment aligner called ALFALFA. BMC Bioinform 16(1):159. 10.1186/s12859-015-0533-0 PMC4449525 25971785

[R101] Williams L, Mumey B (2020) Maximal perfect haplotype blocks with wildcards. iScience 23(6):101149. 10.1016/j.isci.2020.101149 32446220 PMC7243190

